# Using worldwide edaphic data to model plant species niches: An assessment at a continental extent

**DOI:** 10.1371/journal.pone.0186025

**Published:** 2017-10-19

**Authors:** Santiago José Elías Velazco, Franklin Galvão, Fabricio Villalobos, Paulo De Marco Júnior

**Affiliations:** 1 Laboratório de Ecologia Florestal, Departamento de Ciências Agrarias, Universidade Federal do Paraná, Curitiba, Paraná, PR, Brasil; 2 Laboratorio de Macroecología Evolutiva, Red de Biología Evolutiva, Instituto de Ecología, AC, Xalapa, Veracruz, México; 3 Laboratório de Teoria, Metacomunidades e Ecologia de Paisagens, Departamento de Ecologia, ICB, Universidade Federal de Goiás, Goiânia, GO, Brasil; University of Vigo, SPAIN

## Abstract

Ecological niche modeling (ENM) is a broadly used tool in different fields of plant ecology. Despite the importance of edaphic conditions in determining the niche of terrestrial plant species, edaphic data have rarely been included in ENMs of plant species perhaps because such data are not available for many regions. Recently, edaphic data has been made available at a global scale allowing its potential inclusion and evaluation on ENM performance for plant species. Here, we take advantage of such data and address the following main questions: What is the influence of distinct predictor variables (e.g. climatic vs edaphic) on different ENM algorithms? and what is the relationship between the performance of different predictors and geographic characteristics of species? We used 125 plant species distributed over the Neotropical region to explore the effect on ENMs of using edaphic data available from the SoilGrids database and its combination with climatic data from the CHELSA database. In addition, we related these different predictor variables to geographic characteristics of the target species and different ENM algorithms. The use of different predictors (climatic, edaphic, and both) significantly affected model performance and spatial complexity of the predictions. We showed that the use of global edaphic plus climatic variables generates ENMs with similar or better accuracy compared to those constructed only with climate variables. Moreover, the performance of models considering these different predictors, separately or jointly, was related to geographic properties of species records, such as number and distribution range. The large geographic extent, the variability of environments and the different species’ geographical characteristics considered here allowed us to demonstrate that global edaphic data adds useful information for plant ENMs. This is particularly valuable for studies of species that are distributed in regions where more detailed information on soil properties is poor or does not even exist.

## Introduction

Ecological niche and species distribution modeling (ENM and SDM, respectively) are widely-used tool in different fields of plant ecology, including the prediction of new populations of rare species [1]; or potential distribution of invasive species [2]; informing conservation practices for threatened taxa [3,4]; estimating the effect of climatic change on species distributions [5,6]; describing macroecological patterns [7] and studying past species distributions under a paleobiological approach [8]. Despite the broad application of ENMs in botanical studies, debates surrounding several aspects of ENMs continue to date. One of the most important of these aspects relates to the actual data used in ENMs [9].

Data used for conducting ENMs can be grouped in two sets: biogeographical data about the distribution (or presence/absence) of species (i.e. occurrence records) and environmental data (i.e. predictor variables) used to predict those distributions [10]. ENM performance is sensitive to several characteristics of these two datasets [11–21]. For example, regarding biogeographical data, ENMs can be affected by different aspects of the species’ distributional patterns and their sampling such as: prevalence (considered here as the ratio between the quantity of presence and absences), range size and spatial autocorrelation [11,12,22]; which in turn are related to the available sample size [14], data biases along road networks or cities [20,23], geographical accuracy [15] and environmental variability captured by the records [13]. All of these aspects can interact with the environmental data selected to fit the ENMs, and affect model accuracy [21,24] and resultant suitability [19]. Even if occurrence data were bias-free, environmental data can still severely affect ENM performance, especially if inappropriate environmental variables are used as predictors [25].

Choosing a particular environmental variable for ENM depends on the modeling purpose and its biological significance to the species under study [26]. Obviously, different species may have particular constraints related to their dependency on environmental factors and no single variable is expected to be equally meaningful for all species. For instance, variables related to soil properties are considered to be particularly important in determining the distribution of plant species, but have little direct effect on the distribution of the majority of animal species [27]. Considering this plant-soil relationship, predictors can be grouped, following [28], in: (i) resource, matter and energy consumed by an organism, such as oxygen, water, macronutrients and micronutrients; (ii) variables that have direct physiological importance, such as pH, cation exchange capacity, aluminum concentration, hydromorphic condition; and (iii) indirect variables that do not have important physiological effects, such as porosity, bulk density, texture (clay, silt and sand fraction) and soil depth.

In contrast with climatic variables commonly used for ENMs, which describe environmental variation at regional scales (a.k.a. “macroclimatic” variables; e.g. CHELSA; [29]), edaphic variables vary at local scales and with great complexity [30]. For example, within the same landscape, climatic conditions can be very homogenous throughout while soil properties can vary widely according to different parental material [31], topographic position [32] or land-use [33]. Indeed, there are several examples in the literature where soil properties control the distribution of plant species or the structure, composition, and physiognomy of a community within an otherwise climatically homogeneous geographical extent. For instance, mangrove distribution is strongly influenced by soil properties such as salinity, acidity, hydromorphy and nutrient supply [34]. Swamp forests, like the *Caxeitais* (dominated by *Tabebuia cassinoides* (Lam.) DC.) of the Brazilian coast, are mainly distributed over organic and hydromorphic soils [35]. The halophyte vegetation from Chile and Europe is restricted to continental salines [36,37]. Furthermore, soil scarcity can also determine natural plant formations such as those inhabiting rocky outcrops [38]. Narrow plant endemics are also frequently associated with specific types of soil, rock, and bedrock [39]. Even certain soil nutrients can determine the distributional transition from one vegetation type to another, such as that between Neotropical seasonal forests and savannas where the concentration of aluminum or potassium define the structure of these vegetation types [40,41].

Consequently, it is clear that edaphic conditions play an important role in determining the niche of terrestrial plant species [25,42]. Accordingly, several studies have tested the effect of including edaphic variables in ENMs for plant species such as the importance of soil nutritional variables for predicting plant distribution [43]; the improvement of plant ENMs performance when using physical and chemical soil data [21,24]; and the effect of both landscape and edaphic data in predicting future plant distributions under climate change scenarios [9,44]. All of these studies reinforced the idea that plant ENMs could be improved by using a single or a group of edaphic variables. Unfortunately, edaphic variables are still not frequently used as predictors in plant ENMs, which continue to be limited to climatic variables [42]. One reason for this lack of consideration of edaphic variables in plant ENMs may be related to the geographical extent for which these data are available. Such availability has been usually restricted to certain countries or regions (e.g. USA, China or the European Union), whereas in many other regions, as in many Latin America countries, these data are simply not available. Recently, however, the ISRIC World Soil Information with the SoilGrids database has provided data related to physical, chemical and taxonomical characteristics of soils across the globe [45]. Therefore, this database allows the construction of ENMs for plant species inhabiting large regions of the world or species occurring in countries that differ in the quantity and quality of the available edaphic data.

Indeed, despite including detailed soil data, most plant ENM studies have been conducted on extents that are usually smaller than the complete geographic distribution of plant species. Such ENMs may not comprise the full environmental variability that characterizes a species distribution and thus may affect model performance [46,47]. Here, we evaluate the potential effect of using the SoilGrids global dataset in improving ENMs for plant species. We used 125 species distributed along the Neotropical region of the Americas, where many countries do not have detailed soil data, to explore the effect of using global edaphic data and its combination with climatic data in the prediction of models constructed under commonly used ENM algorithms. In addition, we related the different variable sets to certain geographical characteristics of target species (e.g. occurrence area, number of records and density of records) and different algorithms.

## Methods

### Overview

**Fig 1:**
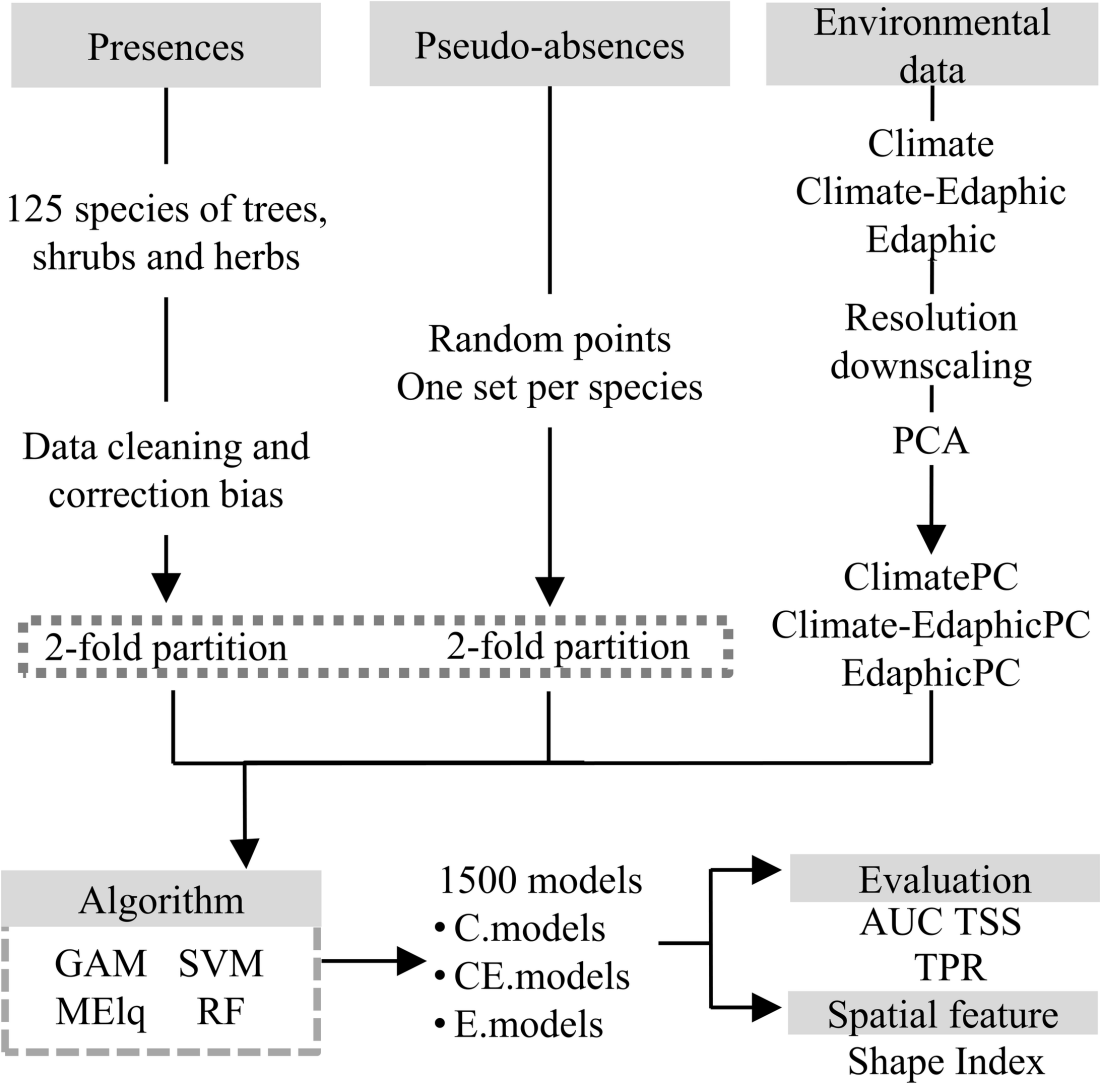
Experimental design for testing the effect of using edaphic variables in ENMs for plant species.

To evaluate the effect of adding global edaphic data into ENMs and its relationship with different modeling algorithms, we adopted a factorial experimental design with two factors: Predictor and Algorithm, with three and four levels respectively, totalizing 12 combinations of factor levels. The first factor, Predictor, comprised three different models based on different predictor sets (edaphic, climatic, or both). For the second factor, Algorithm, we used four types of ENM algorithms (Fig 1). The 12 treatments were applied to 125 plant species, our experimental units, thus 1500 models were fitted (see below).

### Study area

Our study area extended from the south of the United States of America to the austral extremes of Chile and Argentina. This area covers a wide variety of climatic conditions, geological formations, and soil types, but many of its constituent countries lack edaphic data. Consequently, our selected plant species (see below) occur in different biomes, from arid regions such as the *Chihuahuan* and *Caatinga* steppe and warm-humid biomes such as the Brazilian Atlantic and *Chaco-Darién* moist forests to the cold regions of the *Nothofagus* forests and *Andean páramos* (see S1 Table for the complete species list).

### Environmental data

We used three sets of environmental variables for building ENMs: climate-only, edaphic-only and both climatic and edaphic variables together [21,43], hereafter called C.models, E.models and CE.models, respectively. Note that all of these predictors were continuous variables. For the C.models, we employed the 19 bioclimatic variables from the recently developed CHELSA v1.1 online database [29]. These variables were built based on monthly averages of climate data, mainly temperature and precipitation as collected from meteorological stations, for the 1979–2013 period and interpolated to the global surface [29]. E.models were built with 56 variables related to physical and chemical soil properties obtained from the SoilGrids database available from ISRIC-World Soil Information [45], the data were downloaded in June of 2015 (Table 1). The SoilGrids database provides global maps of soil classes and some edaphic variables (see Table 1). In addition, this database has an automated updating system that progressively increases its accuracy when new input data becomes available in the international soil profile databases [45]. The CE.models were built combining the climate and edaphic datasets, summing up to 75 variables. Both climatic and edaphic datasets were acquired with a spatial resolution of 30 arc-seconds (≈ 1 km^2^ cell size) and upscaled to 5 arc-minutes (≈ 10 km^2^ cell size). This upscaling (resolution change) was based on the aggregation, by taking their average value, of lower resolution cells into higher resolution cells. Later, these datasets were cropped to the extent of the study region ranging from -120° to -30° in longitude and -60° to 35° in latitude.

**Table 1:** Climate and edaphic variables (names and units) used as predictors in plant ecological niche models.

Climate (Source: CHELSA)	Unit	Edaphic (Source: SoilGrids)	Unit
Annual Mean Temperature	°C	Depth to bedrock up to maximum 240 cm	cm
Mean Diurnal Range	°C	Predicted probability of occurrence of R horizon	%
Isothermality	°C	Mean of bulk density*	kg/m3
Temperature Seasonality	°C	Mean of coarse fragments volumetric*	%
Max temperature of warmest week	°C	Mean of soil texture fraction clay*	%
Min temperature of coldest week	°C	Mean of soil texture fraction silt*	%
Temperature annual range	°C	Mean of soil texture fraction sand*	%
Mean temperature of wettest quarter	°C	Mean of cation exchange capacity*	cmolc/kg
Mean temperature of driest quarter	°C	Mean of soil organic carbon stock*	Tn/ha
Mean temperature of warmest quarter	°C	Mean of soil organic carbon content*	‰
Mean temperature of coldest quarter	°C	Mean of soil pH in H2O*	
Annual precipitation	Mm		
Precipitation of wettest week	Mm		
Precipitation of driest week	Mm		
Precipitation seasonality	C of V		

*Data for six depths

Different modeling approaches present different sensitivity to collinearity of predictor variables [48]. However, no single methodological procedure has been considered ideal for solving or handling collinearity [48]. Here, we opted to conduct a principal component analysis (PCA) on the original environmental dataset and use the scores of each derived principal components (PCs) as new predictors variables [16,49]. The PCA is a multivariate technique that produces uncorrelated components from the original data sorted according to the amount of total variance that it explains. We selected a number of PCs that explained more than 95% of the total variance in the original dataset [46]. The major advantages of this procedure are the correction of multicollinearity among the original variables, the use of almost all information contained in a large dataset that is captured in the PCs, and the reduction of the number of variables used in the models. Accordingly, C.models and E.models were built with the first six PCs and the first 11 for the CE.models (see S2 Table and S3 Table for more information about variance explained and variables’ coefficients for the selected PCs). The reduced number of new variables (PCs) reveals the high collinearity in our original variable set. In fact, the first two PCs of the PCAs conducted for each variable set explained more than 50% of the variance (in the S1 Fig the relationships of original variables and the first two PCs of each variable set are depicted).

### Plant species data and cleaning

We selected 125 terrestrial plant species distributed within the Neotropical region with the purpose of considering the wide variety of environmental conditions in our study region. Data for these taxa was restricted to the species level, thus infraspecific taxa were not considered. Our final species dataset comprised trees (82), shrubs (27), herbs (8) and palm (8) species. We considered only species with more than 20 checked records (described below; see S1 Table). This dataset comprised species inhabiting extreme latitudes such as *Atriplex canescens*, *Prosopis glandulosa* or *Parthenium incanum* in the north, and *Nothofagus antarctica*, *N*. *pumillo*, and *Mulguraea tridens* in the south. These species also differ in regard to their geographic range sizes, from those with narrow distributions such as *Juglans australis* to those considered as cosmopolites such as *Trema micranta*, *Ipomoea carnea* and *Inga vera*.

We conducted a taxonomic revision for these taxa verifying their accepted names and synonymy using The Plant List Version 1.1. (http://www.theplantlist.org/) and Tropicos (http://www.tropicos.org), checked by the Taxonomic Name Resolution Service v3.2 [50] based on APG III [51]. After confirming accepted names and synonymy, we used these names to search occurrence records for these species in the Global Biodiversity Information Facility (http://www.gbif.org/) and the speciesLink database (http://splink.cria.org.br/).

Occurrence records available in those databases may contain some taxonomic and geographic coordinate errors [52]. Our first step for data cleaning was the elimination of all records allocated outside the study area and those with repeated geographic coordinates. We also removed those species’ records corresponding to invasive or cultivated distributions, thus leaving only those records that pertain to the natural distribution of species. This last step was conducted by using information about species distributions available in the Catalogue of Life (http://www.catalogueoflife.org/), Flora del Conosur (www.darwin.edu.ar/Proyectos/FloraArgentina/fa.htm), List of Species of the Brazilian Flora (http://floradobrasil.jbrj.gov.br/), Smithsonian Tropical Research Institute-Scientific Databases (http://biogeodb.stri.si.edu/bioinformatics/en/), PLANTS Database (http://plants.usda.gov/java/) and Tropicos national species list from Bolivia, Panamá, Paraguay, Peru and Ecuador (http://www.tropicos.org/). In order to clean records temporally, we only considered records that were collected between 1979 and 2013, thus corresponding to the temporal span of our climate variables.

It is common that species’ occurrence records are biased towards roads, cities or countries [23,53,54]. Therefore, these records are not the result of random and homogeneous sampling along the geographic distribution of a species, compromising the accuracy of ENMs [20]. We used a systematic sampling given its suggested effectiveness to correct geographic bias [55] by creating a grid with a resolution of 10 arc min (≈ 20 km^2^ cell size) and then selecting one occurrence per cell. The number of cleaned records for species ranged from 20 to 1227 (see S1 Table).

### Modeling procedures

The diverse algorithms usually employed to build ENMs have different input requirements [56], degrees of complexity [57], stability [58] and predictive abilities [22,59]. For these reasons, we also explored how different algorithms respond to distinct sets of environmental variables. We used four methods which are commonly used in ENM and highlighted for their performance: Generalized Additive Models (GAM), Maximum Entropy (ME), Random Forest (RF) and Support Vector Machine (SVM).

GAM is a non-parametric extension of GLM (Generalized Linear Model) that replaces the linear relationship between the dependent and independent variables by the sum of a smooth function [60]. Owing to its combination of a link function and a smooth function, the GAM method has the ability to deal with highly non-linear and non-monotonic relationships between the response and explanatory variables [61]. These GAMs were fitted using a binomial distribution with all single predictor variables, i.e. without backward or forward selection and interaction. The Newton method was used to optimize the estimation of the smoothing parameter.

ME is a machine learning method based on the principle of maximum entropy [62,63]. This principle is based on minimizing the relative entropy between two probability densities defined in feature space [64]. This is a high performance technique [59] and is less sensitive to spatial errors than others algorithms [65]. This method can be tuned with different features such as linear, quadratic, product, threshold, hinge and binary; the default use of all these features can cause overfitting and affect the models performance [66]. Thus we used linear and quadratic features ([67], hereafter MElq), both of these constrain the approximation of the probability distribution in a way that the variables’ mean and variance should be close to its observed values [62]. We also used 1000 maximum iterations, default regularization values, logistic output format and 10000 maximum background points.

SVM uses linear models to find a decision function, which is a hyperplane determined by non-linear decision boundaries that split samples in different classes within a higher-dimensional space [68,69]. The optimal hyperplane is the one that maximizes the buffer between the boundary (i.e. support vectors) and the data [70]. Mapping of the input data in a high-dimensional feature space is defined by a kernel function [71]. These models were built based on probability classes, performed with a radial basis kernel (RBF) and with a constant cost value (*C* = 1).

RF comprises a family of algorithms that perform classification and regression analyses. RF is a modification of bagging trees, which build a model based on the average of a large collection of non-correlated trees [72]. In each node of these trees, a random sample of *m* predictors is chosen as split candidates from the full set of predictors [73]. These algorithms have the advantage of not overfitting the data [74] and use the out-of-bag (OOB) sample to construct different variable importance measures [72]. To determine the optimal number of variables randomly sampled at each split the RF algorithm was tuned automatically. 500 trees were used at the tuning step, with default values of the step factor and the improvement in OOB error parameter. We considered those models with the minimum OOB error as our final RF models.

Given that we did not have real absences of our species, we created pseudo-absences to fit GAM, SVM and RF models. The prevalence and the method of pseudo-absence allocation can affect ENM performance, which can vary for distinct algorithms [75–77]. To reduce potential noise, we used a prevalence of 1, thus the number of pseudo-absences for each species was equal to its presences. These pseudo-absences were allocated across the study area, which constitutes the biogeographic domain that the modeled species could have used as an accessible area over relevant periods of time [78,79]. We used one soil layer as a raster mask for creating the pseudo-absences given that some cells with climate data may have no soil data (i.e. “empty cells”), such as lakes and some mountain regions.

### Model evaluation

Models were evaluated by a 2-fold cross-validation where the presences of each species and its respective pseudo-absences were partitioned into 50–50% training-testing sets. To control for spatial autocorrelation between training and testing records, we used a checkerboard partitioning method similar to [80]. This method generates checkerboard grids that partition the records into bins by subdividing the geographic extent equally. For this, a particular grid resolution (i.e. cell size) must be chosen a priori, which does not guarantee a balanced number of records in each bin [80]. Therefore, we adapted the method to select the grid resolution that optimizes representation and balance of records within bins. To do so, we created 30 grids with resolutions varying from 0.5 to 15 degrees, with a gradual increase of 0.5. The optimum grid resolution was the one which (i) represented both training and testing records and (ii) minimized the difference between the number of training and testing records. Finally, to maintain a prevalence of 1, we randomly allocated pseudo-absences within each partition group.

Model performance was assessed by dependent and independent threshold metrics [81]. We used the True Positive Rate (TPR) and the True Skill Statistic (TSS) [82] as threshold-dependent indices and the Receiver Operating Curve (AUC) as a threshold-independent evaluation. The threshold was the value that maximized the sum of sensitivity and specificity that produced the most accurate predictions [83]. The complexity of the different spatial patterns of binary predictions (ENM outputs) was evaluated using the shape index (SI). This index measures the complexity of the predicted patches of pixels (i.e. potentially suitable cells) by considering the relationship between the sums of each patch perimeter (*p*_*i*_) divided by the square root of patch area (*a*_*i*_), $\text{SI}=\sum_{1}^{i}\left({0.25\;p}_{i}/\sqrt{a_{i}}\right)$ [84].

### Data analysis

We used Repeated Measures ANOVAs to test the effect of the Predictor (C.models, E.models and CE.models), Algorithm (e.g. GAM, SVM, etc.) and their interaction on TPR, TSS, AUC and SI indices. We assumed that the Predictor and Algorithm as within-subject factors. To perform this analysis correctly and avoid a high Type II error rate, it was necessary that the data met the sphericity condition: the variances of the differences between combinations of levels do not differ. We used Mauchly's Sphericity Test at 95% confidence to validate the sphericity condition of the covariance matrix. When this condition was rejected, the degrees of freedom were corrected by the Greenhouse-Geisser method and used Type III sums of squares. We performed a post-hoc test using linear contrasts based on linear mixed effect models, considering the Predictor and Algorithm as fixed factors and the species as random factor. These models were used to perform pairwise comparisons of means between different predictors for a single algorithm at 95% confidence level. The p-values were corrected using the false discovery rate procedure.

After evaluating the models, their predicted suitabilities were projected onto the geographical space. For each species, we conducted pair-wise comparisons between the suitabilities of different kinds of models and algorithms by calculating the Kendall rank correlation coefficient (τ) with cells of the entire study area. Values of this coefficient range from -1 (perfect disagreement) to 1 (perfect agreement), with values near zero representing independence between the compared ranks.

We used Pearson correlation (*r*) to explore the relationship between variation captured by records for different predictor sets and species’ geographic characteristics, which were, for each species: (i) geographical extent, based on the number of cells within a minimum convex polygon comprising all of a species’ records; (ii) number of records and (iii) density of records, which is the ratio between a species’ number of records and its geographical extent. For each ENM algorithm, we explored the effect of such species’ characteristics and predictors on TSS [12] by fitting linear mixed-effect models. These characteristics were considered as fixed effects within the mixed-effect models, along with the models with different predictors (C.models, E.models and CE.models), whereas the species were considered as random effects. TSS values were arcsine transformed. We used the variance inflation factor (VIF) to test for collinearity among predictors (species geographic characteristics), their significances were determined by a likelihood ratio test.

Construction of ENMs and statistical analyses were conducted in the R environment v. 3.3.2 [85]. The *dismo* v. 1.1.1 package [86] was used to create pseudo-absences, model prediction and validation, and to fit MElq using Maxent v. 3.3.3. The GAMs, SVMs and RF models were fitted using the *gbm* v. 2.1.1 [87], *kernlab* v. 0.9.25 [88] and *randomForest* v. 4.6.12 [89] packages, respectively. We used the packages *raster* v. 2.5.8 [90], *SDMTools* v. 1.1.221 [91], and *pcaPP* v. 1.9.61 [92] to handle raster, calculate the shape index, and the Kendall rank correlation coefficient. To fit the linear mixed effect models, repeated measures ANOVAs and the pairwise mean contrasts, we used the packages *nlme* v. 3.1.128 [93], *lsmeans* 2.26.3 [94] and *car* v. 2.1–5 [95], respectively.

## Results

The use of different predictors (climatic, edaphic, and both) significantly affected model performance, as measured by the TSS, TPR and AUC indices. They also affected the spatial complexity of the geographic predictions (SI). Moreover, TSS, TPR, AUC and SI showed different responses regarding the use of the distinct ENM algorithms. The interactions between predictors and algorithms were significant for TSS, AUC and SI (Table 2).

**Table 2:** Results of the repeated measures ANOVA for the TSS, TPR, AUC and SI, considering the algorithm (GAM, MEql, SVM and RF) and predictor (climate, climate-edaphic, edaphic) factors.

Index	Factors	Sum of Squares	Df	Mean Square	F
TSS	Algorithm	0.577	2.501	0.294	79.429***
	Predictor	1.344	1.463	0.858	76.316***
	Algorithm * Predictor	0.027	4.220	0.006	4.657***
TPR	Algorithm	0.043	2.588	0.021	13.024***
	Predictor	0.242	1.560	0.184	44.654***
	Algorithm * Predictor	0.008	5.119	0.001	1.505^ns^
AUC	Algorithm	0.261	2.035	0.107	76.929***
	Predictor	0.379	1.457	0.249	70.262***
	Algorithm * Predictor	0.014	3.541	0.004	6.013***
SI	Algorithm	17965.610	1.705	10534.815	176.159***
	Predictor	77863.060	1.487	52370.852	690.234***
	Algorithm * Predictor	2286.069	4.684	488.070	47.693***

Degrees of freedom were corrected using Greenhouse-Geisser estimate of sphericity. TSS: true skill statistic; TPR: true positive rate, AUC: area under curve; SI: shape index.

Significance

*** *P* < 0.001

** *P* < 0.01

* *P* < 0.05

^ns^ *P* > 0.05

**Fig 2:**
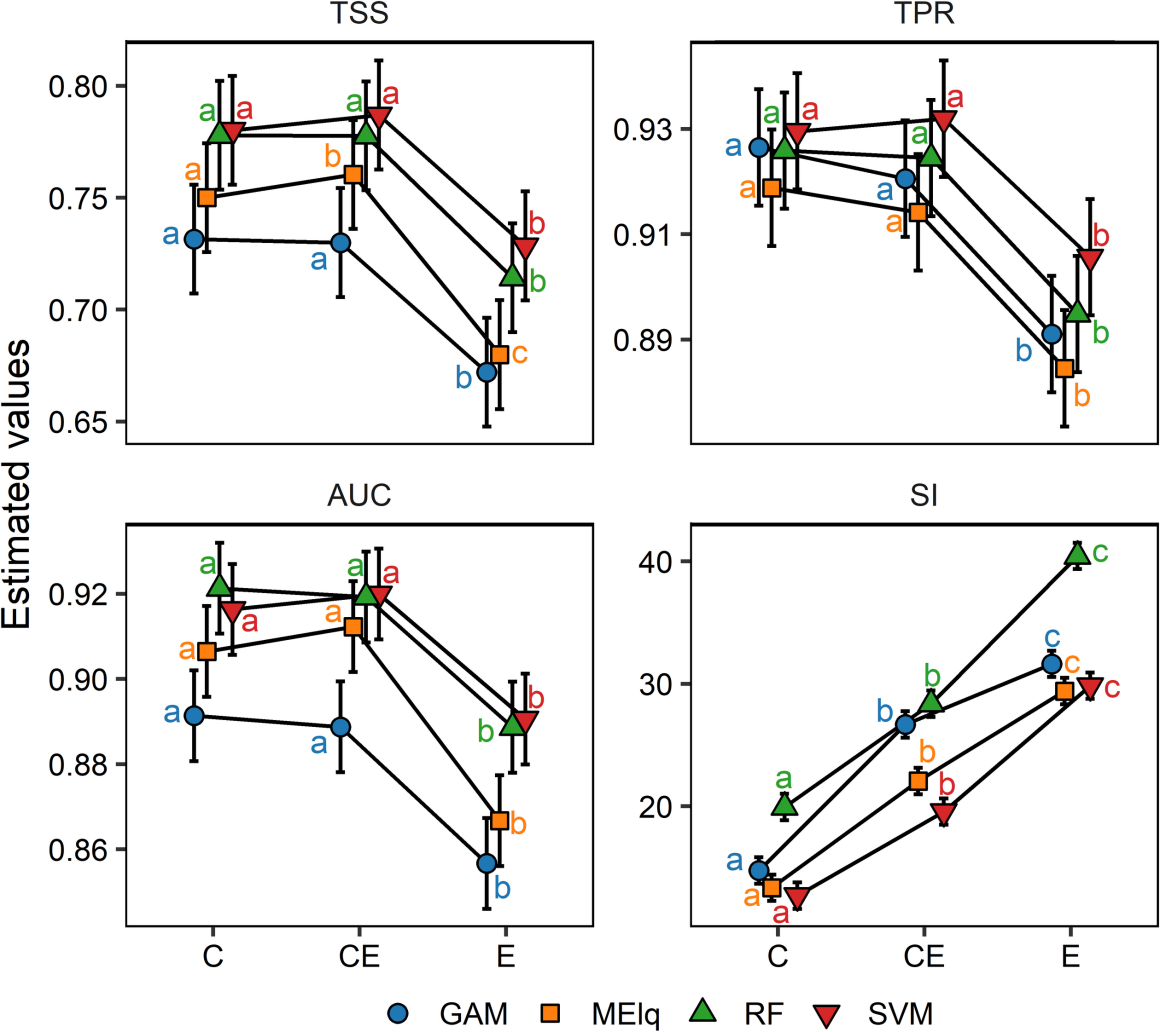
Estimated values and confidence interval (bars) for TSS, AUC, TPR and SI of models fitted with three set of predictors combined with four algorithms. Means with same letter for different predictor and same algorithm denote significant difference using the linear contrast (P < 0.05). TSS: true skill statistic, TPR: true positive rate, AUC: area under curve; SI: shape index, C: models with climate predictors, CE: models with climate and edaphic predictors, E: models with edaphic predictors.

According to TSS, TPR, and AUC, C.models and CE.models performed better than E.models, regardless of the ENM algorithm used. Nonetheless, MElq performed better for the CE.models, regarding TSS, whereas SVM, GAM and RF did not show differences between C.models and CE.models. These results were different for the sensitivity, given that the CE.models showed the best values for SVM. Moreover, no algorithm differed significantly regarding only the C.models (Fig 2A). Regardless of the predictor set, the SVM and RF algorithms had the highest values of TSS and AUC, followed by MElq and GAM. Regarding the spatial complexity of predictions, C.models showed the most aggregated and continuous prediction, whereas the E.models had the most spread and complex patterns. The CE.models had an intermediate shape complexity. Independent of the predictor set, RF created the most complex spatial patterns, whereas SVM showed the lowest SI (Fig 2A).

**Fig 3:**
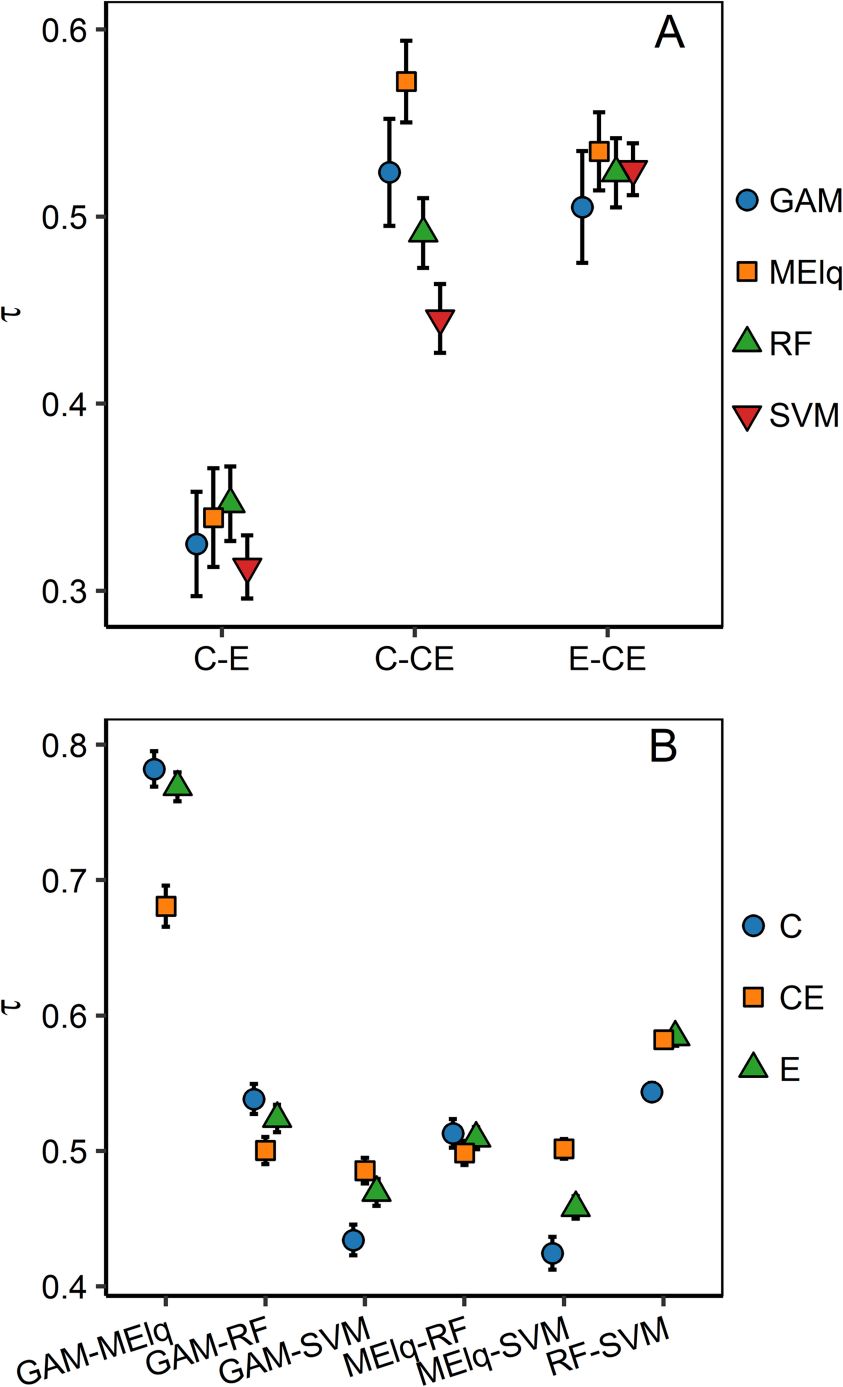
Mean and confidence interval of Kendall rank correlation coefficient (τ) of pair comparison of suitability. (A) Suitability comparison between models with predictors sets for different algorithm. (B) Suitability comparison between algorithms for different predictors sets. C: models with climate predictors, CE: models with climate and edaphic predictors, E: models with edaphic predictors.

Mean values of Kendal rank correlation of suitabilities always showed positive values for the pair-wise comparison of models with different predictors (Fig 3A). The highest values of suitability correlation were for C.models-CE.models and E.models-CE.models for all algorithms. MElq had the most similar suitability for these paired comparisons. The lowest correlation was between the suitability of C.models and E.models, with mean values smaller than 0.4 for all algorithms. For the C.models-E.models comparison, the highest correlations were for MElq and RF, whereas in the C.models-CE.models comparison, highest correlation was for MElq and GAM, and for the E.models-CE.models comparison highest correlations were for MElq, RF and SVM (Fig 3A). These comparisons of suitability between algorithms showed that GAM-MElq were the most similar, followed by RF-SVM, for any predictor set. The lowest correlation was found between GAM-SVM and between MElq-SVM (Fig 3B).

The relationship between different geographic characteristics of species revealed that species with wider distributions had also more records sampled (*r* = 0.790, *p* < 0.001) but they showed lower record density (*r* = -0.640, *p* < 0.001). However, the relationship between number of records and their density was weak (*r* = -0.090, *p* < 0. 334) (S2 Fig). Widely distributed species presented higher standard deviation for the first principal component of the climate predictors (*r* = 0.670, *p* < 0.001). These patterns were weaker for the climate-edaphic (*r* = 0.240, *p* = 0.007) and edaphic predictors set (*r* = 0.130, *p* = 0.146) (S3 Fig; S4 Table).

The linear mixed-effect models revealed, for all algorithms, that the species geographical extent negatively affected the TSS, the number of records affected SVM, whereas for this algorithm number of records affected the TSS positively, implying better model performance (S4 Fig). In addition, we found that different predictors affected model accuracy but the interaction among predictors and species geographic characteristic differed among the ENM algorithms. Interaction between predictor sets and geographical extent were significant for GAM, MElq and RF, but not for SVM, which had significant interaction between the number of records and predictor sets. (Table 3; S4 Fig). Species geographic characteristics, predictor sets, and their interaction explained between 54 and 68% of model performance (TSS) variability (Table 3).

**Table 3:** Summary of linear mixed effect models for four algorithms and the significance of covariates. The model selection was based on the likelihood ratio test.

Algorithm	Covariates	LRT χ^2^	Df	p-value	R^2^
GAM	GE	110.204	1	<0.001	
	NR	0.081	1	0.777	
	DR	0.001	1	0.969	
	Predictor	63.803	2	<0.001	
	GE*Predictor	21.418	2	<0.001	
	NR*Predictor	5.704	2	0.058	
	DR*Predictor	0.628	2	0.628	54.255
MElq	GE	110.405	1	<0.001	
	NR	2.104	1	0.147	
	DR	0.003	1	0.957	
	Predictor	12.642	2	0.002	
	GE*Predictor	10.736	2	0.005	
	NR*Predictor	4.654	2	0.097	
	DR*Predictor	0.191	2	0.909	68.686
RF	GE	138.093	1	<0.001	
	NR	0.424	1	0.515	
	DR	0.387	1	0.534	
	Predictor	139.853	2	<0.001	
	GE*Predictor	2.678	2	0.262	
	NR*Predictor	3.558	2	0.169	
	DR*Predictor	1.876	2	0.391	64.059
SVM	GE	59.592	1	<0.001	
	NR	19.461	1	<0.001	
	DR	0.281	1	0.596	
	Predictor	133.931	2	<0.001	
	GE*Predictor	4.389	2	0.111	
	NR*Predictor	19.322	2	<0.001	
	DR*Predictor	0.233	2	0.890	68.614

GE: geographical extent; NR: Number of records; DR: density of records; Predictor: models constructed with climate, climate-edaphic or edaphic variables; Df: degree of freedom; LRT χ^2^: Chi square for the likelihood radio test. R^2^: marginal determination coefficient calculated for the final models with significance values < 0.05 of their covariates.

**Fig 4:**
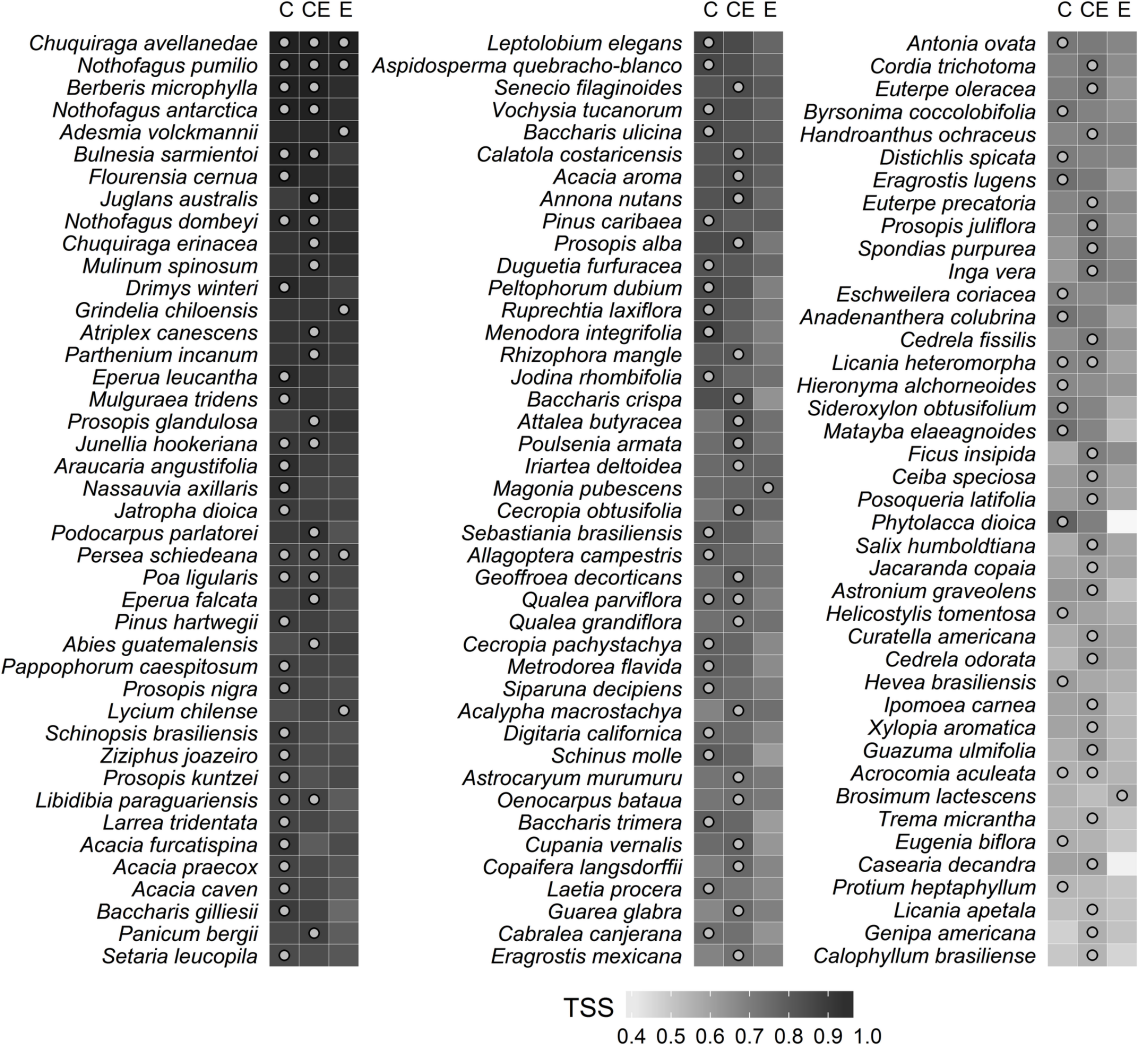
List of modelled species and model accuracy performed with SVM and three predictors sets. Species are sorted by their overall TSS mean, the points represent the highest model accuracy for a particular species. TSS: true skill statistic, C: models with climate predictors, CE: models with climate and edaphic predictors, E: models with edaphic predictors.

**Fig 5:**
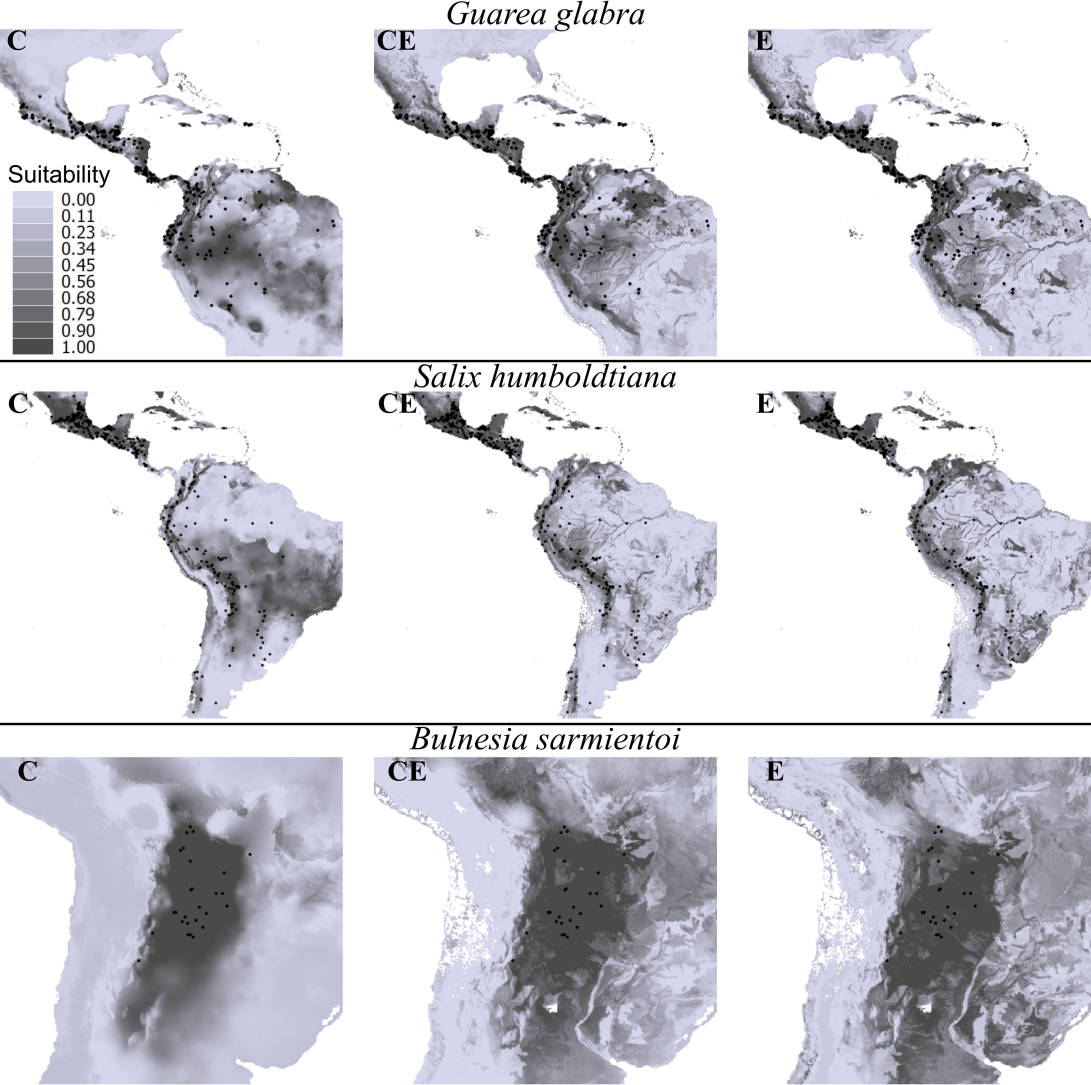
Examples of change in suitability predicted by SVM models for three species derived from the use of three predictors set. C: models with climate predictors, CE: models with climate and edaphic predictors, E: models with edaphic predictors.

As expected, the response of the ENMs to different predictors varied individually for each species. Thus, for some species, the use of edaphic data (E.models and CE.models) considerably improved model accuracy in comparison with those models constructed with climate-only predictors (e.g. *Astronium graveolens*, *Cedrela odorata*, *Ficus insipida*, *Genipa Americana*, *Guarea glabra* and *Salix humboldtiana*). Conversely, edaphic-only predictors notably decreased model performance for other species (e.g. *Casearia decandra*, *Phytolacca dioica*, *Hevea brasiliensis*, *Matayba eleagnoides*, *Schinus molle* and *Baccharis crispa*). In addition, there were species that presented similar outputs irrespective of the kind of predictors used for modeling (e.g. *Chuquiraga avellanedae*, *Nothofagus pumilio* and *Persea schiedeana*; Fig 4). The effect of different predictor variables on the species’ suitability pattern varied among species. For example, for species such as *Salix chilensis* and *Guarea glabra* that have broad distributions, CE.models and E.models showed suitable areas that were more constrained compared to those from C.models. Conversely, *Bulnesia sarmientoy* showed an expansion of the suitable areas for those models that used edaphic data compared to those that did not include these data (Fig 5).

Based on SVM models, better performance when using the CE.models was observed for 54 species, whereas for 53 species this was true when using the C.models and five species showed the best model performance when using E.models. Also, there were species whose models had the same maximum accuracy independent of the predictors set used. For example, models with climatic-only or climatic-edaphic predictors performed equally well for 13 species, whereas edaphic-only or climatic-edaphic predictors did the same for three species. Finally, models for only three species showed the same TSS irrespective of the considered predictor variables (Fig 5).

## Discussion

We have shown here the advantages of using worldwide edaphic data as predictors in ecological niche models for plant species. More specifically, we showed that ENMs constructed with commonly used climatic variables plus edaphic variables did not affect negatively the performance of ENMs but instead improved the accuracy for some algorithms. This happened even when the ENMs based only on edaphic variables did not provide accurate predictions for any algorithm. Owing to the particular spatial patterning of these different predictor sets, climatic and edaphic, their use also affected the shape and spatial complexity of ENM outputs. In addition, the performance of models considering these predictor sets, separately or jointly, was strongly related to geographic properties of species records, irrespective of the algorithm. Our findings highlight the feasibility and advantages of including global soil data, along with climatic variables, into ENMs to achieve accurate predictions of plant species distributions.

Soils are the consequence of different forming factors such as climate, organisms, topography, parent material, time [96], among other local factors [97]. Any particular combination of these factors will give rise to particular processes that can be extremely complex [97,98], involving disintegration, integration, weathering, decomposition, neoformation and transformation [99]. These factors and processes acting in soil genesis define the chemical and physical characteristics of soils, which will ultimately determine the underground environment for terrestrial plants. We want to reinforce here the widely accepted idea that soil is one of the most important factors affecting plant ecology and that the common practice of only using climatic predictors represents a weak conceptual basis for the application of ENMs for plant species (see also [25]). Despite potential disadvantages of our testing framework, such as the lack of real species absences and the extension of the edaphic data used, we demonstrated that the combination of climatic and global edaphic predictor variables increases the accuracy of ENMs for several of our studied plant species. In fact, our findings are consistent with previous studies that found similar results for plants at smaller extents and finer resolutions [9,21,43,44].

The type of organisms under study must guide the selection of predictor variables for ENMs and SDMs. Accordingly, soil properties should be considered when applying ENMs for plant species, whereas these properties may be neglected when modeling animal species [27]. However, few studies doing ENM or SDM with plant species have used variables related to soil or, for that matter, variables different than climatic ones [42,100]. Adding or excluding variables when describing the species environmental niches can affect the form of the resulting hypervolume in multivariate space (S5 Fig) [101]. Owing to the reciprocity between the environmental and geographic space [102], changes in the multivariate space can affect ENM predictions on geographic space. In our case, this fact may be responsible for the low consensus between the suitability patterns (Fig 3A) and the geometry of predicted maps (Fig 2) of our modeled species. Regardless of the ENM algorithm used (e.g. GAM vs MElq), using edaphic predictors alone or jointly with climatic variables (E.models and CE.models, respectively) augmented the spatial complexity of the predicted plant distributions. This results from the complex spatial variation of soil properties [30] in comparison with climatic variables. Indeed, the spatial complexity of edaphic variables could be responsible for the lower TSS and AUC values predicted by our models based only on edaphic predictors, which are consistent with results obtained for several plant species in Canada with models constructed with the same type of variable and more detailed edaphic data [21]. Given that different algorithms or variables could produce models with the same accuracy but with different spatial predictions (see Fig 5; [103]), we highlight the importance of acknowledging that model evaluation must be based both on an accuracy metric (e.g. TSS, AUC, etc.) and a preliminary visual examination based on the ecological knowledge of the studied species and their relationships with the selected predictors [26].

It is well known that the performance of an ENM algorithm can vary according to the characteristics of the species niche [104], the training dataset [16,77,105], and how the algorithm is tuned [66,106]. One reason that could explain the discrepancy on the accuracy of our different algorithms is the prevalence between presence and absences. For instance, ENMs built under SVM and RF produce better models when the presences/absences ratio is 1, whereas GAM models achieve higher performances with lower presences/absences ratio values [107]. In our case, the fact that we used a number of pseudo-absences equal to that of presences for all algorithms can have negatively affected GAM compared to the other algorithms. Nevertheless, the high positive correlation between GAM and MElq suitabilities is noteworthy. Such correlation may result from the ME tuning with linear and quadratic terms, a feature that reduces the complexity of the ME model making it more similar to GAM. Despite ME being characterized as a high-performance algorithm [59], here this method was outperformed by SVM and RF even when it was conducted with 10,000 background points instead of using the pseudo-absences as in the other algorithms [106]. This finding may be particular for ENMs of plant species, for which SVM and RF have been referred as two of the most accurate algorithms for modeling Neotropical plants [108]. In addition, we found that SVM and RF were the most accurate methods, although producing different predictions, a fact that reflects their ability to represent complex non-linear relationships. Moreover, each one of these algorithms has important advantages; RF constructs models that avoid overfitting [74] whereas SVM has the ability to construct stable models even with a large set of covariates [108]. In fact, both algorithms were the best classifiers with the UC Irvine Machine Learning Repository [109].

The geographic range size of species can influence the performance of ENMs [11,12,22]. For instance, small-ranged species could have a limited variability of environmental conditions captured by its presences [110] and results from their ENMs may be more marginal (i.e. the difference between the mean environmental condition of the species and the mean of the study areas [111]) in comparison to other widely distributed species within a particular study region. Our study supports this interpretation given that narrowly distributed species had lower climatic variation represented by their occurrence records. This pattern is more evident in models that considered climatic variables only whereas models including edaphic variables showed wide variability and low correlation between environmental variation and species’ range-size (S3 Fig). This could explain why some species with the largest geographic ranges showed increased accuracy with the use of the edaphic predictors, whereas for many of the restricted species similar accuracy was observed when using climatic variables only or climatic and edaphic variables together. This finding stresses the dissimilar nature of climatic and edaphic variables and the different way in which those predictors interact with the geographic characteristics of species records.

Our results also showed that species with wide geographic distributions and large numbers of occurrences produce models with lower accuracy, a tendency that is consistent with previous findings [11,12,22,105], whereas species with a denser aggregation of records showed the opposite trend (S4 Fig). On this point we agree with [112], in that the relationship between model accuracy and the geographic range of a species is strongly affected by the extent used to construct the models, which is consequently related with the relative occurrence area and marginality of species. In addition, different extents and resolutions can influence the relative importance of predictors and the predicted suitability [113]. As the extent increases for an individual model, the environmental difference between predicted and unpredicted cells may increase simply by the broader environmental variability captured by larger extents. This may inflate the metrics designed to estimate model accuracy [114], especially for species distributed in marginal areas of the environmental space. Other causes for inflating the estimated model accuracy is the relationship between the number of occurrence records and accuracy. In our case, widely distributed species turned out to be more sampled (i.e. had more records) but, as mentioned above, species with more records also showed lower accuracies (S2 Fig). Again, one simple explanation for this is that a low discrimination of the environment between predicted and unpredicted cells is expected for species with large distributional area [11].

To the extent of our knowledge, this is the first attempt to evaluate the reliability of using global edaphic information to perform ENMs of plant species over large regions. Nevertheless, ENMs/SDMs experiments have some practical limitations because these approaches are sensible by several factors such as the extent of the area used to construct the models [115], the covariates selected and their grain [21,113], the geographical characteristics and quality of the species records [12,22], the pseudo-absences allocation methods [77], the algorithms used and their tuning [106] or even the species selected to perform the experiments [110], we acknowledge that there is no “silver bullet” approach that is capable of dealing with all those potential situations [104]. Therefore, we suggest that all comparative modeling studies such as ours need to be extrapolated with care. Of course, we are aware that the edaphic data we used may have some deficiencies related to the information on soil sampling and covariates used to generate these data [45] and that it is difficult to make generalizations to other regions of the world. However, the large geographic extent, the variability of environments and the different species geographic characteristics considered here allowed us to show that such global edaphic data adds useful information for plant distribution modeling. This is particularly valuable for studies of species that are distributed in regions where more detailed information on soil properties is poor or does not even exist. Importantly, we do not imply that these global edaphic data must be used in all future studies applying ENMs for plant species, but we do encourage modelers to test some of these edaphic variables and evaluate their model outputs against those conducted with climatic variables only. Recently the SoilGrids was improved by using more accurate technics and with finer-resolution data [116], thus we suggest that future studies consider the effect of different resolutions of soil data when applied to plant ENMs.

## Supporting information

S1 Fig Ordination diagram for the first two axis of three PCAs conducted with three variable set. C: models with climate predictors, CE: models with climate and edaphic predictors, E: models with edaphic predictors.
(TIF)

S2 Fig Relationship between geographical extent, number of records and density of records for the 125 target species. (TIFF)

S3 Fig Relationship between the standard deviation for the first principal component, of three predictors set, captured by the records of each species and their relationship with geographical extent, number of records and density of records. C: models with climate predictors, CE: models with climate and edaphic predictors, E: models with edaphic predictors.
(TIFF)

S4 Fig Effect of geographical extent, number of records and density of records on the TSS for GAM, MElq, RF and SVM conducted with three predictors sets. TSS: this index was transformed to arcsine; C: models with climate predictors; CE: models with climate and edaphic predictors; E: models with edaphic predictors.
(TIFF)

S5 Fig Predicted suitability by the SVM method for *Cedrella odorata* and its relationship between the geographical and environmental space for three predictors sets. The right panel shows the first two principal components of the PCA conducted for each variable set. C: models with climate predictors, CE: models with climate and edaphic predictors, E: models with edaphic predictors.
(TIFF)

S1 Table List of species modeled, families, habit and number of cleaned record (NR). (PDF)

S2 Table Principal components selected from the PCAs, their eigenvalues, variance explained and cumulative variance explained for each variable set. (PDF)

S3 Table Coefficients of the principal components selected from the PCAs performed for each dataset. (PDF)

S4 Table List of species modeled, number of records (NR), geographical extent (GR), density of records (DP) and standard deviation of the first principal component of climatic variables only (C.SD), climatic and edaphic variables (CE.SD) and edaphic variables only (E.SD). (PDF)

S1 File Principal components of climate variables used to perform the climate-only models. The data are in GeoTIFF format.
(RAR)

S2 File Principal components of edaphic variables used to perform edaphic-only models. The data are in GeoTIFF format compressed.
(RAR)

S3 File Principal components of climatic and edaphic variables used to perform climatic and edaphic models. The data are in GeoTIFF format compressed.
(RAR)
