# Supplementary figures and images for: Using worldwide edaphic data to model plant species niches: An assessment at a continental extent
Source: PLoS One. 2017 Oct 19;12(10):e0186025. doi: 10.1371/journal.pone.0186025 (PMC5648144; doi:10.1371/journal.pone.0186025)

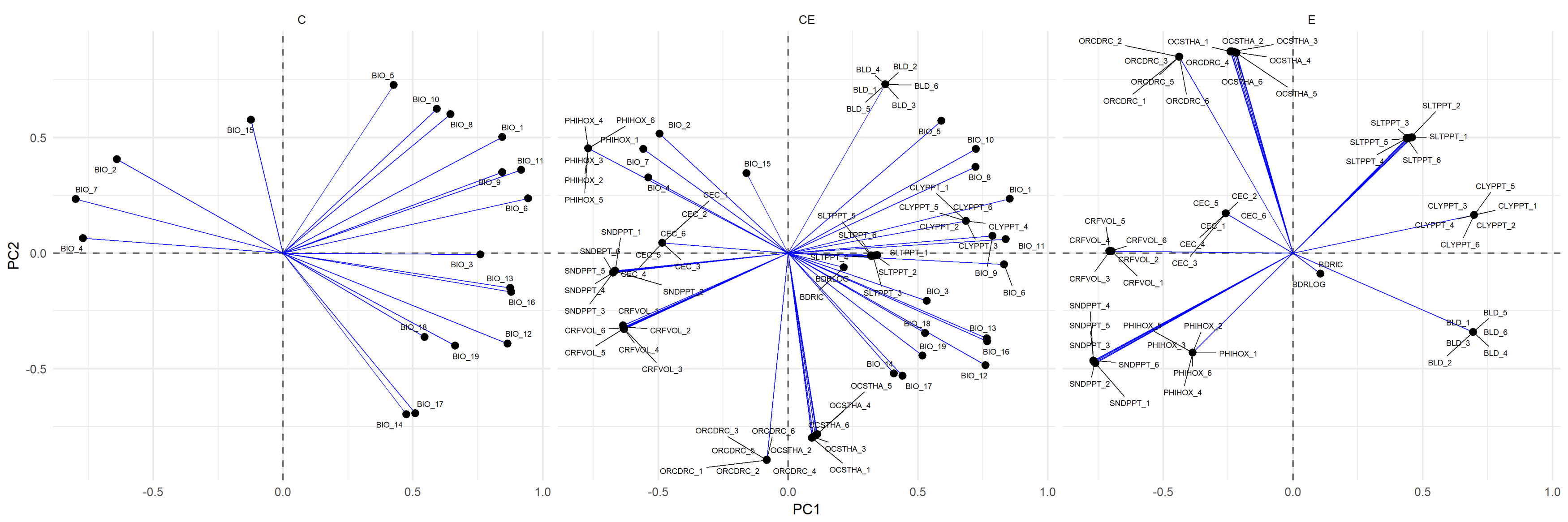

Supplement: S1 Fig — C: models with climate predictors, CE: models with climate and edaphic predictors, E: models with edaphic predictors. (TIF) [file pone.0186025.s001.tif]

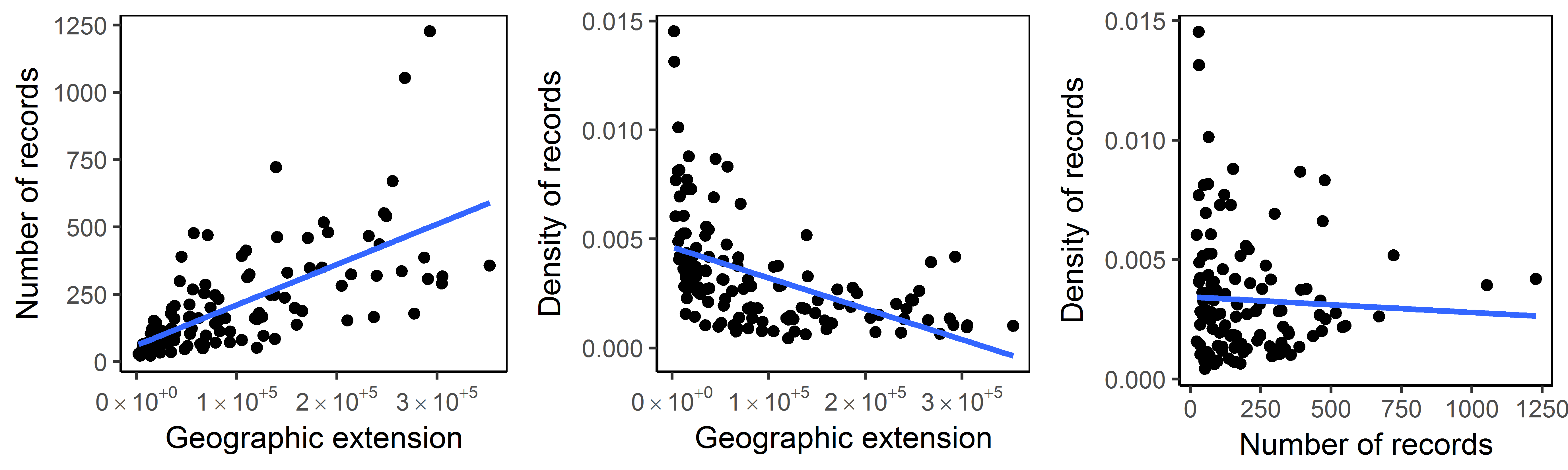

Supplement: S2 Fig — (TIFF) [file pone.0186025.s002.tiff]

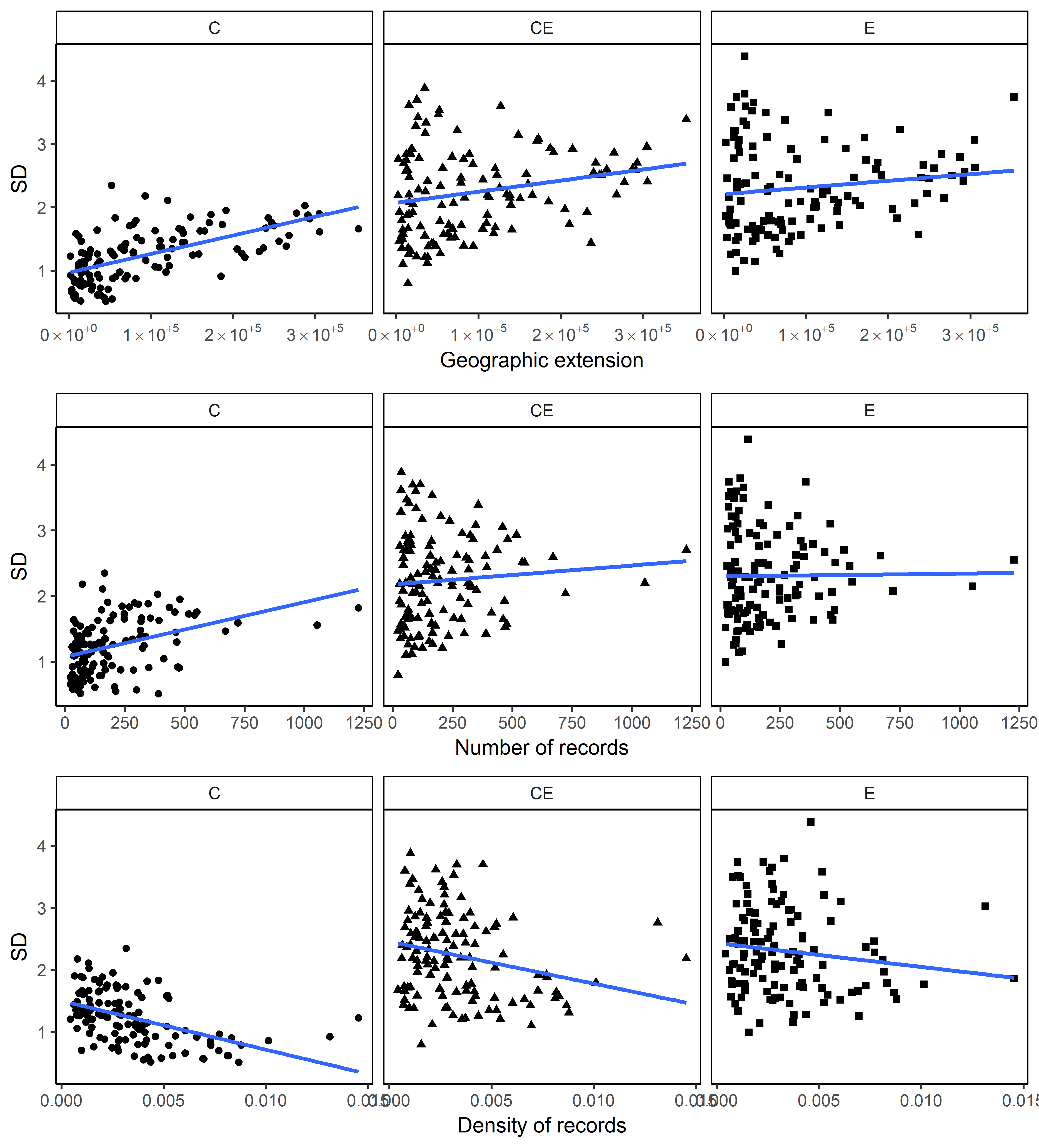

Supplement: S3 Fig — C: models with climate predictors, CE: models with climate and edaphic predictors, E: models with edaphic predictors. (TIFF) [file pone.0186025.s003.tiff]

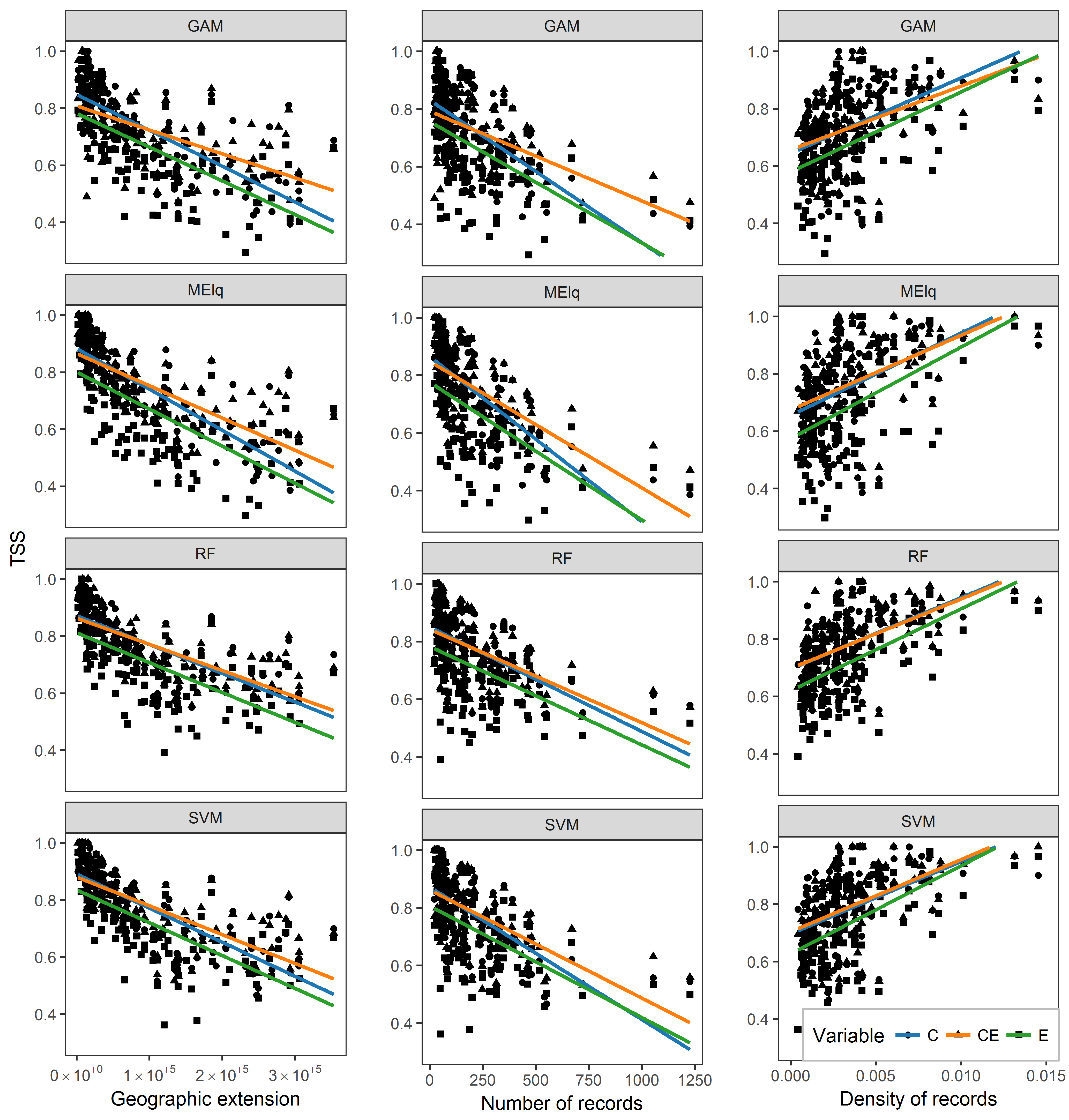

Supplement: S4 Fig — TSS: this index was transformed to arcsine; C: models with climate predictors; CE: models with climate and edaphic predictors; E: models with edaphic predictors. (TIFF) [file pone.0186025.s004.tiff]

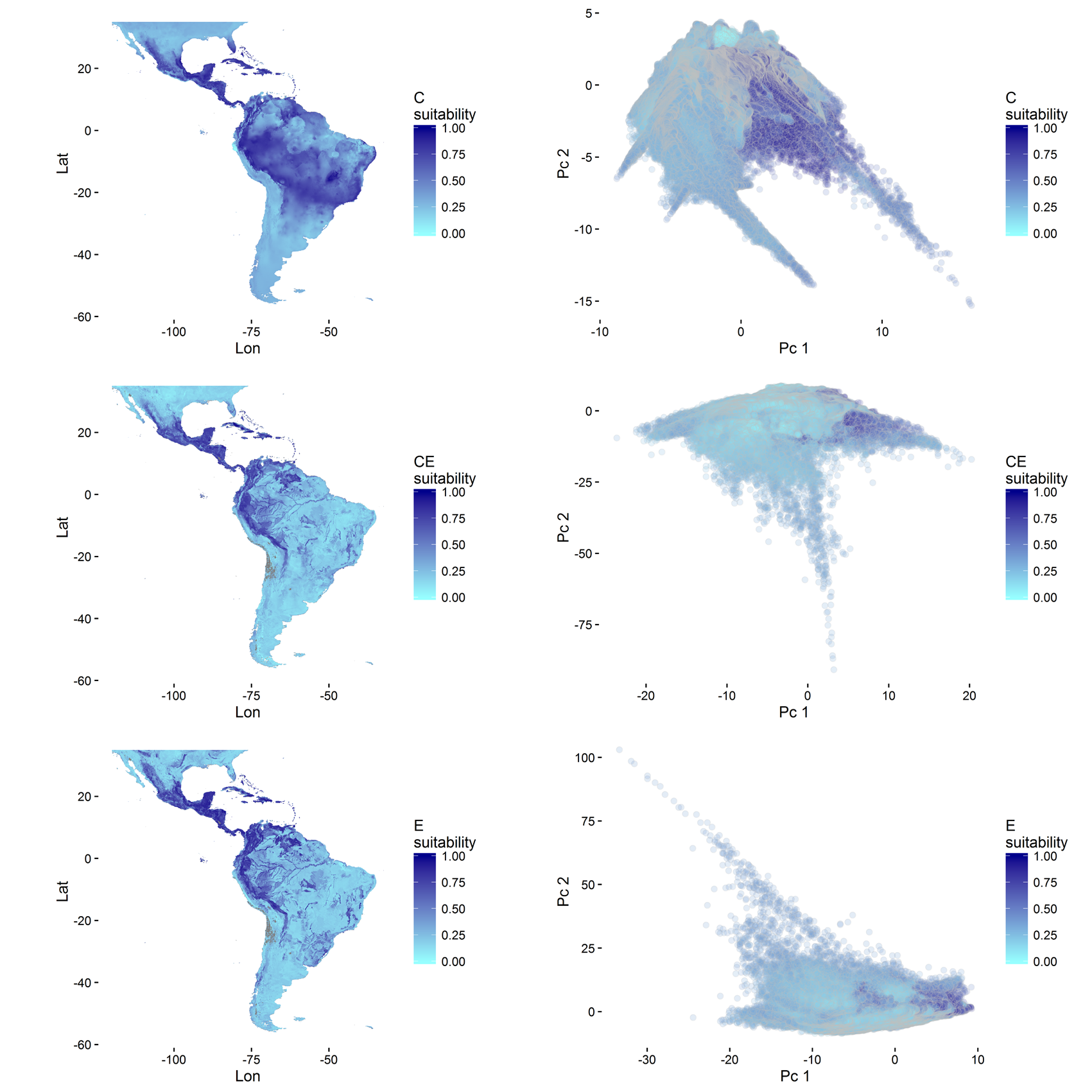

Supplement: S5 Fig — The right panel shows the first two principal components of the PCA conducted for each variable set. C: models with climate predictors, CE: models with climate and edaphic predictors, E: models with edaphic predictors. (TIFF) [file pone.0186025.s005.tiff]
