# Supplementary material for: Using worldwide edaphic data to model plant species niches: An assessment at a continental extent
Source: PLoS One. 2017 Oct 19;12(10):e0186025. doi: 10.1371/journal.pone.0186025 (PMC5648144; doi:10.1371/journal.pone.0186025)
Supplement: S1 Table — (PDF) [file pone.0186025.s006.pdf]

S1 Table. List of species modeled, families, habit and number of cleaned records (NR).

Family	Species	Habit	NR
Pinaceae	*Abies guatemalensis* Rehd.	Tree	46
Fabaceae	*Acacia aroma* Hook. & Arn.	Tree	159
Fabaceae	*Acacia caven* (Molina) Molina	Tree	123
Fabaceae	*Acacia furcatispina* Burkart	Shrub	35
Fabaceae	*Acacia praecox* Griseb.	Tree	75
Euphorbiaceae	*Acalypha macrostachya* Jacq.	Tree	459
Arecaceae	*Acrocomia aculeata* (Jacq.) Lodd. ex Mart.	Palm	166
Fabaceae	*Adesmia volckmannii Phil.*	Shrub	63
Arecaceae	*Allagoptera campestris* (Mart.) Kuntze	Palm	106
Fabaceae	*Anadenanthera colubrina* (Vell.) Brenan	Tree	330
Annonaceae	*Annona nutans* (R.E.Fr.) R.E.Fr.	Shrub	64
Loganiaceae	*Antonia ovata* Pohl	Tree	113
Araucariaceae	*Araucaria angustifolia* (Bertol.) Kuntze	Tree	48
Apocynaceae	*Aspidosperma quebracho-blanco* Schltdl.	Tree	83
Arecaceae	*Astrocaryum murumuru* Mart.	Palm	66
Anacardiaceae	*Astronium graveolens* Jacq.	Tree	282
Amaranthaceae	*Atriplex canescens* (Pursh) Nutt.	Shrub	178
Arecaceae	*Attalea butyracea* (Mutis ex L.f.) We	Palm	104
Asteraceae	*Baccharis crispa* Spreng.	Shrub	142
Asteraceae	*Baccharis gilliesii* A.Gray	Shrub	34
Asteraceae	*Baccharis trimera* DC.	Shrub	162
Asteraceae	*Baccharis ulicina* Hook. & Arn.	Shrub	63
Berberidaceae	*Berberis microphylla* Forst.	Shrub	44
Moraceae	*Brosimum lactescens* (S.Moore) C.C.Berg	Tree	248
Zygophyllaceae	*Bulnesia sarmientoi* Lorentz ex Griseb.	Tree	22
Malpighiaceae	*Byrsonima coccolobifolia* Kunth	Shrub	112
Meliaceae	*Cabralea canjerana* (Vell.) Mart.	Tree	157
Icacinaceae	*Calatola costaricensis* Standl.	Tree	201
Calophyllaceae	*Calophyllum brasiliense* Cambess.	Tree	541
Salicaceae	*Casearia decandra* Jacq.	Tree	188
Urticaceae	*Cecropia obtusifolia* Bertol.	Tree	165
Urticaceae	*Cecropia pachystachya* Trécul	Tree	167
Meliaceae	*Cedrela fissilis* Vell.	Tree	137
Meliaceae	*Cedrela odorata* L.	Tree	436
Malvaceae	*Ceiba speciosa* (A. St.-Hil.) Ravenna	Shrub	80
Asteraceae	*Chuquiraga avellanedae* Lorentz	Shrub	35
Asteraceae	*Chuquiraga erinacea* D.Don	Shrub	75
Fabaceae	*Copaifera langsdorffii* Desf.	Tree	233
Boraginaceae	*Cordia trichotoma* (Vell.) Steud.	Tree	286
Sapindaceae	*Cupania vernalis* Cambess.	Tree	97
Dilleniaceae	*Curatella americana* L.	Tree	467
Poaceae	*Digitaria californica* (Benth.) Henrard	Herb	324

Family	Species	Habit	NR
Poaceae	*Digitaria californica* (L.) Greene	Herb	357
Winteraceae	*Drimys winteri* J.R.Forst. & G.Forst.	Tree	53
Annonaceae	*Duguetia furfuracea* (A.St.-Hil.) Saff.	Tree	390
Fabaceae	*Eperua falcata* Aubl.	Tree	72
Fabaceae	*Eperua leucantha* Benth.	Tree	29
Poaceae	*Eragrostis lugens* Nees	Herb	336
Poaceae	*Eragrostis mexicana* (Hornem.) Link	Herb	387
Lecythidaceae	*Eschweilera coriacea* (DC.) S.A.Mori	Tree	324
Myrtaceae	*Eugenia biflora* (L.) DC.	Tree	200
Arecaceae	*Euterpe oleracea* Mart.	Palm	72
Arecaceae	*Euterpe precatoria* Mart.	Palm	393
Moraceae	*Ficus insipida* Willdenow	Tree	480
Asteraceae	*Flourensia cernua* DC.	Shrub	55
Rubiaceae	*Genipa americana* L.	Tree	551
Fabaceae	*Geoffroea decorticans* (Hook. & Arn.) Burkart	Tree	58
Asteraceae	*Grindelia chiloensis* (Cornel.) Cabrera	Shrub	43
Meliaceae	*Guarea glabra* Vahl	Tree	238
Malvaceae	*Guazuma ulmifolia* Lam.	Tree	1054
Bignoniaceae	*Handroanthus ochraceus* (Cham.) Mattos	Tree	162
Moraceae	*Helicostylis tomentosa* (Poepp. & Endl.) J.F.Macbr.	Tree	248
Euphorbiaceae	*Hevea brasiliensis* (Willd. ex A.Juss.) Müll.Arg.	Tree	85
Phyllanthaceae	*Hieronyma alchorneoides* Allemão	Tree	347
Fabaceae	*Inga vera* Willd.	Tree	670
Convolvulaceae	*Ipomoea carnea* Jacq.	Shrub	317
Arecaceae	*Iriartea deltoidea* Ruiz & Pav.	Palm	268
Bignoniaceae	*Jacaranda copaia* (Aubl.) D.Don	Tree	315
Euphorbiaceae	*Jatropha dioica* Sessé	Shrub	120
Santalaceae	*Jodina rhombifolia* Hook. & Arn. ex Reissek	Tree	22
Juglandaceae	*Juglans australis* Griseb.	Tree	29
Verbenaceae	*Junellia hookeriana* (Covas & Schnack) N. O'Leary & P. Peralta	Shrub	77
Salicaceae	*Laetia procera* (Poepp.) Eichler	Tree	167
Zygophyllaceae	*Larrea tridentata* (DC.) Coville	Shrub	350
Fabaceae	*Leptolobium elegans* Vogel	Tree	207
Fabaceae	*Libidibia paraguariensis* (D. Parodi) G.P. Lewis	Tree	105
Chrysobalanaceae	*Licania apetala* (E.Mey.) Fritsch	Tree	314
Chrysobalanaceae	*Licania heteromorpha* Benth.	Tree	413
Solanaceae	*Lycium chilense* Bert.	Shrub	97
Sapindaceae	*Magonia pubescens* A.St.-Hil.	Tree	79
Sapindaceae	*Matayba elaeagnoides* Radlk.	Tree	50
Oleaceae	*Menodora integrifolia* Steud.	Shrub	36
Rutaceae	*Metrodorea flavida* K. Krause	Tree	91
Verbenaceae	*Mulguraea tridens* (Lag.) N.O'Leary & P.Peralta	Shrub	32
Apiaceae	*Mulinum spinosum* Pers.	Shrub	144
Asteraceae	*Nassauvia axillaris* (Lag. ex Spreng.) D.Don	Shrub	73

Family	Species	Habit	NR
Nothofagaceae	*Nothofagus antarctica* (G.Forst.) Oerst.	Tree	43
Nothofagaceae	*Nothofagus dombeyi* (Mirb.) Oerst.	Tree	30
Nothofagaceae	*Nothofagus pumilio* (Poepp. & Endl.) Krasser	Tree	30
Arecaceae	*Oenocarpus bataua* Mart.	Palm	247
Poaceae	*Panicum bergii* Arechav.	Herb	124
Poaceae	*Pappophorum caespitosum* R.E.Fr.	Herb	83
Asteraceae	*Parthenium incanum* Kunth	Shrub	152
Fabaceae	*Peltophorum dubium* (Spreng.) Taub.	Tree	124
Lauraceae	*Persea schiedeana* Nees	Tree	45
Phytolaccaceae	*Phytolacca dioica* L	Tree	52
Pinaceae	*Pinus caribaea* Morelet	Tree	61
Pinaceae	*Pinus hartwegii* Lindl.	Tree	64
Poaceae	*Poa ligularis* Nees ex Steud.	Herb	115
Podocarpaceae	*Podocarpus parlatorei* Pilg.	Tree	65
Rubiaceae	*Posoqueria latifolia* (Rudge) Schult.	Tree	517
Moraceae	*Poulsenia armata* (Miq.) Standl.	Tree	152
Fabaceae	*Prosopis alba* Griseb.	Tree	75
Fabaceae	*Prosopis glandulosa* Torr.	Tree	197
Fabaceae	*Prosopis juliflora* (Sw.) DC.	Tree	178
Fabaceae	*Prosopis kuntzei* Kuntze	Tree	36
Fabaceae	*Prosopis nigra* Hieron.	Tree	54
Burseraceae	*Protium heptaphyllum* (Aubl.) Marchand	Tree	722
Vochysiaceae	*Qualea grandiflora* Mart.	Tree	470
Vochysiaceae	*Qualea parviflora* Mart.	Tree	477
Rhizophoraceae	*Rhizophora mangle* L.	Tree	307
Polygonaceae	*Ruprechtia laxiflora* Meisn.	Tree	71
Salicaceae	*Salix humboldtiana* Willd.	Tree	290
Anacardiaceae	*Schinopsis brasiliensis* Engl.	Tree	212
Anacardiaceae	*Schinus molle* L.	Tree	96
Euphorbiaceae	*Sebastiania brasiliensis* Spreng.	Tree	162
Asteraceae	*Senecio filaginoides* DC.	Shrub	68
Poaceae	*Setaria leucopila* (Scribn. & Merr.) K.Schum.	Herb	181
Sapotaceae	*Sideroxylon obtusifolium* (Roem. & Schult.) T.D.Penn.	Tree	153
Siparunaceae	*Siparuna decipiens* (Tul.) A.DC.	Shrub	255
Anacardiaceae	*Spondias purpurea* L.	Tree	319
Cannabaceae	*Trema micrantha* (L.) Blume	Tree	1227
Vochysiaceae	*Vochysia tucanorum* Mart.	Tree	299
Annonaceae	*Xylopia aromatica* (Lam.) Mart.	Tree	462
Rhamnaceae	*Ziziphus joazeiro* Mart.	Tree	47
