# Supplementary material for: Using worldwide edaphic data to model plant species niches: An assessment at a continental extent
Source: PLoS One. 2017 Oct 19;12(10):e0186025. doi: 10.1371/journal.pone.0186025 (PMC5648144; doi:10.1371/journal.pone.0186025)
Supplement: S2 Table — (PDF) [file pone.0186025.s007.pdf]

S2 Table. Principal components selected from the PCAs, their eigenvalues, variance explained and cumulative variance explained for each variable set.

Set of Variables	Principal Components	Eigenvalues	Variance explained for each PC	Cumulative variance explained
**Climate**	1	9.419	49.600	49.600
	2	4.129	21.700	71.300
	3	2.481	13.100	84.400
	4	0.933	4.900	89.300
	5	0.780	4.100	93.400
	6	0.423	2.200	95.600
**Edaphic**	1	16.284	29.078	29.078
	2	13.852	24.736	53.814
	3	11.629	20.767	74.581
	4	5.935	10.598	85.179
	5	4.336	7.742	92.922
	6	1.741	3.109	96.031
**Climate and Edaphic**	1	21.959	29.300	29.300
	2	16.614	22.200	51.400
	3	12.910	17.200	68.600
	4	6.770	9.000	77.700
	5	4.783	6.400	84.100
	6	3.448	4.600	88.600
	7	1.790	2.400	91.000
	8	1.279	1.700	92.700
	9	1.158	1.500	94.300
	10	1.053	1.400	95.700
	11	0.803	1.100	96.800
