# Supplementary material for: Using worldwide edaphic data to model plant species niches: An assessment at a continental extent
Source: PLoS One. 2017 Oct 19;12(10):e0186025. doi: 10.1371/journal.pone.0186025 (PMC5648144; doi:10.1371/journal.pone.0186025)
Supplement: S3 Table — (PDF) [file pone.0186025.s008.pdf]

S3 Table. Coefficients of the Principal components selected from the PCAs performed for each variable set.

CLIMATE

Variable	PC1	PC2	PC3	PC4	PC5	PC6
BIO_1	0.269	0.256	-0.096	0.064	-0.073	0.017
BIO_2	-0.204	0.206	-0.067	-0.495	0.087	0.481
BIO_3	0.242	-0.003	0.333	-0.054	-0.064	0.517
BIO_4	-0.245	0.033	-0.395	-0.022	0.165	-0.111
BIO_5	0.136	0.37	-0.345	-0.027	0.1	0.043
BIO_6	0.301	0.12	0.064	0.182	-0.079	-0.006
BIO_7	-0.254	0.119	-0.316	-0.23	0.161	0.037
BIO_8	0.206	0.306	-0.184	-0.065	-0.194	0.043
BIO_9	0.269	0.178	-0.017	0.164	0.102	0.054
BIO_10	0.189	0.318	-0.328	0.079	0.024	-0.035
BIO_11	0.292	0.183	0.064	0.064	-0.104	0.054
BIO_12	0.275	-0.2	-0.079	-0.209	0.138	-0.105
BIO_13	0.279	-0.077	0.062	-0.309	0.244	-0.29
BIO_14	0.152	-0.355	-0.286	0	-0.106	0.355
BIO_15	-0.039	0.293	0.384	-0.386	0.119	0.097
BIO_16	0.28	-0.086	0.059	-0.306	0.24	-0.276
BIO_17	0.162	-0.353	-0.285	-0.005	-0.079	0.319
BIO_18	0.174	-0.185	-0.152	-0.483	-0.528	-0.203
BIO_19	0.211	-0.204	-0.064	0.07	0.638	0.164

EDAPHIC

Variable	PC1	PC2	PC3	PC4	PC5	PC6
BDRIC	0.026	-0.024	0.156	-0.022	-0.044	-0.469
BDRLOG	0.026	-0.024	0.156	-0.022	-0.044	-0.469
BLD_1	0.172	-0.092	-0.055	-0.207	0.014	0.198
BLD_2	0.172	-0.092	-0.055	-0.207	0.014	0.198
BLD_3	0.172	-0.092	-0.055	-0.207	0.014	0.198
BLD_4	0.172	-0.092	-0.055	-0.207	0.014	0.198
BLD_5	0.172	-0.092	-0.055	-0.207	0.014	0.198
BLD_6	0.172	-0.092	-0.055	-0.207	0.014	0.198
CEC_1	-0.064	0.046	-0.256	0.043	-0.132	-0.052
CEC_2	-0.064	0.046	-0.256	0.043	-0.132	-0.052
CEC_3	-0.064	0.046	-0.256	0.043	-0.132	-0.052
CEC_4	-0.064	0.046	-0.256	0.043	-0.132	-0.052
CEC_5	-0.064	0.046	-0.256	0.043	-0.132	-0.052
CEC_6	-0.064	0.046	-0.256	0.043	-0.132	-0.052
CLYPPT_1	0.173	0.044	0.03	0.226	-0.188	0.084
CLYPPT_2	0.173	0.044	0.03	0.226	-0.188	0.084
CLYPPT_3	0.173	0.044	0.03	0.226	-0.188	0.084
CLYPPT_4	0.173	0.044	0.03	0.226	-0.188	0.084
CLYPPT_5	0.173	0.044	0.03	0.226	-0.188	0.084
CLYPPT_6	0.173	0.044	0.03	0.226	-0.188	0.084

Variable	PC1	PC2	PC3	PC4	PC5	PC6
CRFVOL_1	-0.173	0.002	-0.083	0.18	0.188	0.151
CRFVOL_2	-0.174	0.002	-0.078	0.183	0.185	0.15
CRFVOL_3	-0.175	0.002	-0.074	0.185	0.182	0.148
CRFVOL_4	-0.175	0.002	-0.074	0.185	0.182	0.147
CRFVOL_5	-0.175	0.002	-0.075	0.184	0.183	0.148
CRFVOL_6	-0.175	0.002	-0.077	0.183	0.184	0.149
OCSTHA_1	-0.06	0.234	0.043	-0.115	-0.088	0.104
OCSTHA_2	-0.059	0.234	0.044	-0.116	-0.09	0.102
OCSTHA_3	-0.058	0.234	0.044	-0.118	-0.093	0.1
OCSTHA_4	-0.056	0.234	0.046	-0.121	-0.098	0.096
OCSTHA_5	-0.055	0.233	0.048	-0.124	-0.102	0.092
OCSTHA_6	-0.054	0.233	0.049	-0.125	-0.105	0.088
ORCDRC_1	-0.109	0.228	0.04	-0.067	-0.051	0.02
ORCDRC_2	-0.109	0.228	0.04	-0.067	-0.051	0.02
ORCDRC_3	-0.109	0.228	0.04	-0.067	-0.051	0.02
ORCDRC_4	-0.109	0.228	0.04	-0.067	-0.051	0.02
ORCDRC_5	-0.109	0.228	0.04	-0.067	-0.051	0.02
ORCDRC_6	-0.108	0.228	0.04	-0.067	-0.052	0.02
PHIHOX_1	-0.096	-0.116	-0.199	-0.076	-0.162	-0.026
PHIHOX_2	-0.096	-0.116	-0.199	-0.076	-0.162	-0.026
PHIHOX_3	-0.096	-0.116	-0.199	-0.076	-0.162	-0.026
PHIHOX_4	-0.096	-0.116	-0.199	-0.076	-0.162	-0.026
PHIHOX_5	-0.096	-0.116	-0.199	-0.076	-0.162	-0.026
PHIHOX_6	-0.096	-0.116	-0.199	-0.076	-0.162	-0.026
SLTPPT_1	0.114	0.134	-0.167	-0.063	0.198	-0.102
SLTPPT_2	0.112	0.134	-0.168	-0.064	0.199	-0.104
SLTPPT_3	0.11	0.134	-0.169	-0.065	0.2	-0.106
SLTPPT_4	0.109	0.134	-0.17	-0.065	0.201	-0.108
SLTPPT_5	0.109	0.133	-0.17	-0.065	0.202	-0.108
SLTPPT_6	0.109	0.133	-0.17	-0.064	0.202	-0.108
SNDPPT_1	-0.189	-0.128	0.109	-0.085	-0.038	0.028
SNDPPT_2	-0.189	-0.127	0.108	-0.088	-0.034	0.027
SNDPPT_3	-0.19	-0.126	0.107	-0.091	-0.029	0.025
SNDPPT_4	-0.19	-0.125	0.106	-0.094	-0.025	0.024
SNDPPT_5	-0.191	-0.125	0.105	-0.095	-0.024	0.024
SNDPPT_6	-0.191	-0.125	0.105	-0.095	-0.025	0.024

CLIMATE AND EDAPHIC

Variable	PC1	PC2	PC3	PC4	PC5	PC6	PC7	PC8	PC9	PC10	PC11
BIO_1	0.18	0.058	0.037	-0.012	0.014	0.181	0.155	-0.079	-0.054	-0.12	0.088
BIO_2	-0.104	0.126	-0.052	-0.022	-0.012	0.016	-0.185	0.259	0.013	-0.291	0.269
BIO_3	0.113	-0.051	0.092	0.157	-0.068	0.229	0.1	-0.04	0.068	0.182	0.166
BIO_4	-0.113	0.08	-0.108	-0.144	0	-0.236	-0.079	0.009	-0.159	-0.265	-0.077
BIO_5	0.124	0.14	-0.025	-0.091	0.025	0.082	0.11	-0.036	-0.136	-0.431	0.062

Variable	PC1	PC2	PC3	PC4	PC5	PC6	PC7	PC8	PC9	PC10	PC11
BIO_6	0.175	-0.012	0.07	0.048	0.013	0.191	0.192	-0.128	-0.013	0.028	0.007
BIO_7	-0.117	0.11	-0.098	-0.118	0.002	-0.165	-0.147	0.123	-0.078	-0.328	0.035
BIO_8	0.152	0.091	-0.001	-0.059	0.036	0.16	0.136	0	-0.057	-0.148	0.273
BIO_9	0.166	0.018	0.049	0.022	-0.034	0.187	0.149	-0.142	-0.056	-0.129	-0.107
BIO_10	0.153	0.11	-0.015	-0.092	0.015	0.085	0.149	-0.098	-0.151	-0.304	0.046
BIO_11	0.177	0.015	0.067	0.043	0.011	0.22	0.15	-0.067	0.015	-0.006	0.087
BIO_12	0.16	-0.119	0.025	0.065	-0.093	-0.056	0.106	0.089	-0.038	-0.104	0.085
BIO_13	0.161	-0.09	0.035	0.084	-0.08	0.09	0.033	0.132	0.119	-0.176	0.023
BIO_14	0.086	-0.128	-0.021	0.001	-0.097	-0.256	0.195	0.023	-0.354	0.123	0.177
BIO_15	-0.034	0.085	0.018	0.045	0.003	0.376	-0.145	0.175	0.336	-0.052	0.122
BIO_16	0.162	-0.094	0.041	0.082	-0.08	0.078	0.038	0.137	0.119	-0.178	0.027
BIO_17	0.093	-0.13	-0.019	0.004	-0.099	-0.252	0.188	0.019	-0.345	0.101	0.162
BIO_18	0.111	-0.085	-0.025	0.017	-0.049	-0.104	0.052	0.317	-0.033	0.183	0.598
BIO_19	0.109	-0.109	0.028	0.057	-0.108	-0.05	0.165	-0.11	-0.138	-0.26	-0.301
BDRIC	0.045	-0.015	0.12	-0.076	0.045	-0.287	0.062	-0.435	0.347	-0.063	0.205
BDRLOG	0.045	-0.015	0.12	-0.076	0.045	-0.287	0.062	-0.435	0.347	-0.063	0.205
BLD_1	0.079	0.179	-0.046	-0.157	-0.07	0.087	-0.113	-0.119	-0.111	0.135	0.025
BLD_2	0.079	0.179	-0.046	-0.157	-0.07	0.087	-0.113	-0.119	-0.111	0.135	0.025
BLD_3	0.079	0.179	-0.046	-0.157	-0.07	0.087	-0.113	-0.119	-0.111	0.135	0.025
BLD_4	0.079	0.179	-0.046	-0.157	-0.07	0.087	-0.113	-0.119	-0.111	0.135	0.025
BLD_5	0.079	0.179	-0.046	-0.157	-0.07	0.087	-0.113	-0.119	-0.111	0.135	0.025
BLD_6	0.079	0.179	-0.046	-0.157	-0.07	0.087	-0.113	-0.119	-0.111	0.135	0.025
CEC_1	-0.102	0.011	-0.208	0.082	0.1	0.055	0.201	-0.025	-0.003	0.054	-0.036
CEC_2	-0.102	0.011	-0.208	0.082	0.1	0.055	0.201	-0.025	-0.003	0.054	-0.036
CEC_3	-0.102	0.011	-0.208	0.082	0.1	0.055	0.201	-0.025	-0.003	0.054	-0.036
CEC_4	-0.102	0.011	-0.208	0.082	0.1	0.055	0.201	-0.025	-0.003	0.054	-0.036
CEC_5	-0.102	0.011	-0.208	0.082	0.1	0.055	0.201	-0.025	-0.003	0.054	-0.036
CEC_6	-0.102	0.011	-0.208	0.082	0.1	0.055	0.201	-0.025	-0.003	0.054	-0.036
CLYPPT_1	0.144	0.034	-0.032	0.152	0.252	-0.049	-0.127	-0.02	-0.053	0	0.009
CLYPPT_2	0.144	0.034	-0.032	0.152	0.252	-0.049	-0.127	-0.02	-0.053	0	0.009
CLYPPT_3	0.144	0.034	-0.032	0.152	0.252	-0.049	-0.127	-0.02	-0.053	0	0.009
CLYPPT_4	0.144	0.034	-0.032	0.152	0.252	-0.049	-0.127	-0.02	-0.053	0	0.009
CLYPPT_5	0.144	0.034	-0.032	0.152	0.252	-0.049	-0.127	-0.02	-0.053	0	0.009
CLYPPT_6	0.144	0.034	-0.032	0.152	0.252	-0.049	-0.127	-0.02	-0.053	0	0.009
CRFVOL_1	-0.134	-0.077	-0.01	0.212	-0.132	0.083	-0.13	-0.16	-0.113	-0.067	0.091
CRFVOL_2	-0.133	-0.078	-0.006	0.213	-0.128	0.08	-0.131	-0.167	-0.114	-0.064	0.092
CRFVOL_3	-0.133	-0.08	-0.003	0.213	-0.125	0.078	-0.131	-0.173	-0.115	-0.061	0.092
CRFVOL_4	-0.133	-0.08	-0.002	0.213	-0.124	0.077	-0.131	-0.174	-0.114	-0.06	0.091
CRFVOL_5	-0.133	-0.08	-0.004	0.213	-0.125	0.078	-0.13	-0.171	-0.114	-0.06	0.091
CRFVOL_6	-0.133	-0.079	-0.005	0.212	-0.127	0.08	-0.13	-0.168	-0.113	-0.061	0.091
OCSTHA_1	0.019	-0.196	-0.081	-0.157	0.086	0.095	-0.068	-0.05	-0.05	-0.012	0.01
OCSTHA_2	0.02	-0.196	-0.082	-0.159	0.087	0.094	-0.067	-0.047	-0.049	-0.013	0.012
OCSTHA_3	0.021	-0.195	-0.081	-0.161	0.089	0.093	-0.065	-0.044	-0.047	-0.014	0.013
OCSTHA_4	0.022	-0.193	-0.081	-0.165	0.093	0.091	-0.061	-0.041	-0.045	-0.016	0.015
OCSTHA_5	0.023	-0.193	-0.08	-0.168	0.097	0.089	-0.058	-0.039	-0.043	-0.017	0.017

Variable	PC1	PC2	PC3	PC4	PC5	PC6	PC7	PC8	PC9	PC10	PC11
OCSTHA_6	0.024	-0.192	-0.079	-0.171	0.099	0.087	-0.056	-0.038	-0.042	-0.018	0.02
ORCDRC_1	-0.017	-0.219	-0.067	-0.107	0.06	0.05	-0.046	-0.027	0.02	-0.019	0.035
ORCDRC_2	-0.017	-0.219	-0.067	-0.107	0.06	0.05	-0.046	-0.027	0.02	-0.019	0.035
ORCDRC_3	-0.017	-0.219	-0.067	-0.107	0.06	0.05	-0.046	-0.027	0.02	-0.019	0.035
ORCDRC_4	-0.017	-0.219	-0.067	-0.107	0.06	0.05	-0.046	-0.027	0.02	-0.019	0.035
ORCDRC_5	-0.017	-0.219	-0.067	-0.107	0.06	0.05	-0.046	-0.027	0.02	-0.019	0.035
ORCDRC_6	-0.017	-0.219	-0.067	-0.108	0.06	0.05	-0.046	-0.027	0.019	-0.019	0.036
PHIHOX_1	-0.162	0.111	-0.084	-0.032	0.093	0.022	0.072	-0.044	0.006	-0.037	0.129
PHIHOX_2	-0.162	0.111	-0.084	-0.032	0.093	0.022	0.072	-0.044	0.006	-0.037	0.129
PHIHOX_3	-0.162	0.111	-0.084	-0.032	0.093	0.022	0.072	-0.044	0.006	-0.037	0.129
PHIHOX_4	-0.162	0.111	-0.084	-0.032	0.093	0.022	0.072	-0.044	0.006	-0.037	0.129
PHIHOX_5	-0.162	0.111	-0.084	-0.032	0.093	0.022	0.072	-0.044	0.006	-0.037	0.129
PHIHOX_6	-0.162	0.111	-0.084	-0.032	0.093	0.022	0.072	-0.044	0.006	-0.037	0.129
SLTPPT_1	0.072	-0.002	-0.229	-0.008	-0.197	-0.03	-0.005	-0.004	0.108	-0.005	-0.011
SLTPPT_2	0.071	-0.003	-0.229	-0.008	-0.199	-0.03	-0.004	-0.004	0.108	-0.006	-0.012
SLTPPT_3	0.069	-0.003	-0.229	-0.008	-0.201	-0.031	-0.002	-0.004	0.108	-0.007	-0.013
SLTPPT_4	0.068	-0.003	-0.23	-0.008	-0.202	-0.032	0	-0.003	0.107	-0.009	-0.013
SLTPPT_5	0.067	-0.003	-0.23	-0.007	-0.202	-0.033	0	-0.003	0.107	-0.011	-0.013
SLTPPT_6	0.068	-0.002	-0.23	-0.007	-0.202	-0.034	0	-0.003	0.106	-0.013	-0.013
SNDPPT_1	-0.14	-0.019	0.192	-0.084	0.001	0.051	0.078	0.015	-0.05	0.003	0.003
SNDPPT_2	-0.141	-0.019	0.191	-0.086	-0.004	0.052	0.079	0.016	-0.048	0.004	0.003
SNDPPT_3	-0.141	-0.02	0.19	-0.087	-0.009	0.053	0.079	0.018	-0.045	0.006	0.003
SNDPPT_4	-0.142	-0.02	0.189	-0.089	-0.014	0.054	0.08	0.019	-0.042	0.007	0.003
SNDPPT_5	-0.142	-0.021	0.188	-0.089	-0.015	0.054	0.08	0.019	-0.041	0.007	0.003
SNDPPT_6	-0.142	-0.021	0.188	-0.09	-0.014	0.054	0.08	0.018	-0.042	0.008	0.003

Meaning of variable codes

Code	Variable
BIO_1	Annual Mean Temperature
BIO_2	Mean Diurnal Range
BIO_3	Isothermality
BIO_4	Temperature Seasonality
BIO_5	Max Temperature of Warmest Month
BIO_6	Min Temperature of Coldest Month
BIO_7	Temperature Annual Range
BIO_8	Mean Temperature of Wettest Quarter
BIO_9	Mean Temperature of Driest Quarter
BIO_10	Mean Temperature of Warmest Quarter
BIO_11	Mean Temperature of Coldest Quarter
BIO_12	Annual Precipitation
BIO_13	Precipitation of Wettest Month
BIO_14	Precipitation of Driest Month
BIO_15	Precipitation Seasonality
BIO_16	Precipitation of Wettest Quarter

Code	Variable
BIO_17	Precipitation of Driest Quarter
BIO_18	Precipitation of Warmest Quarter
BIO_19	Precipitation of Coldest Quarter
BDRLOG	Predicted probability of occurrence of R horizon OK
BDRIC	Depth to bedrock (R horizon) up to maximum 240 cm OK
BLD_1	Bulk density in kg / cubic-meter for 2.5 cm depth
BLD_2	Bulk density in kg / cubic-meter for 10 cm depth
BLD_3	Bulk density in kg / cubic-meter for 22.5 cm depth
BLD_4	Bulk density in kg / cubic-meter for 45 cm depth
BLD_5	Bulk density in kg / cubic-meter for 80 cm depth
BLD_6	Bulk density in kg / cubic-meter for 150 cm depth
CEC_1	Cation exchange capacity in cmolc/kg for 2.5 cm depth
CEC_2	Cation exchange capacity in cmolc/kg for 10 cm depth
CEC_3	Cation exchange capacity in cmolc/kg for 22.5 cm depth
CEC_4	Cation exchange capacity in cmolc/kg for 45 cm depth
CEC_5	Cation exchange capacity in cmolc/kg for 80 cm depth
CEC_6	Cation exchange capacity in cmolc/kg for 150 cm depth
CLYPPT_1	Soil texture fraction clay in percent for 2.5 cm depth
CLYPPT_2	Soil texture fraction clay in percent for 10 cm depth
CLYPPT_3	Soil texture fraction clay in percent for 22.5 cm depth
CLYPPT_4	Soil texture fraction clay in percent for 45 cm depth
CLYPPT_5	Soil texture fraction clay in percent for 80 cm depth
CLYPPT_6	Soil texture fraction clay in percent for 150 cm depth
CRFVOL_1	Coarse fragments volumetric in percent for 2.5 cm depth
CRFVOL_2	Coarse fragments volumetric in percent for 10 cm depth
CRFVOL_3	Coarse fragments volumetric in percent for 22.5 cm depth
CRFVOL_4	Coarse fragments volumetric in percent for 45 cm depth
CRFVOL_5	Coarse fragments volumetric in percent for 80 cm depth
CRFVOL_6	Coarse fragments volumetric in percent for 150 cm depth
OCSTHA_1	Soil organic carbon stock in tonnes per ha for 2.5 cm depth
OCSTHA_2	Soil organic carbon stock in tonnes per ha for 10 cm depth
OCSTHA_3	Soil organic carbon stock in tonnes per ha for 22.5 cm depth
OCSTHA_4	Soil organic carbon stock in tonnes per ha for 45 cm depth
OCSTHA_5	Soil organic carbon stock in tonnes per ha for 80 cm depth
OCSTHA_6	Soil organic carbon stock in tonnes per ha for 150 cm depth
ORCDRC_1	Soil organic carbon content (fine earth fraction) in permilles for 2.5 cm depth
ORCDRC_2	Soil organic carbon content (fine earth fraction) in permilles for 10 cm depth
ORCDRC_3	Soil organic carbon content (fine earth fraction) in permilles for 22.5 cm depth
ORCDRC_4	Soil organic carbon content (fine earth fraction) in permilles for 45 cm depth
ORCDRC_5	Soil organic carbon content (fine earth fraction) in permilles for 80 cm depth
ORCDRC_6	Soil organic carbon content (fine earth fraction) in permilles for 150 cm depth
PHIHOX_1	Soil pH x 10 in H2O for 2.5 cm depth
PHIHOX_2	Soil pH x 10 in H2O for 10 cm depth
PHIHOX_3	Soil pH x 10 in H2O for 22.5 cm depth
PHIHOX_4	Soil pH x 10 in H2O for 45 cm depth

Code	Variable
PHIHOX_5	Soil pH x 10 in H2O for 80 cm depth
PHIHOX_6	Soil pH x 10 in H2O for 150 cm depth
SLTPPT_1	Soil texture fraction silt in percent for 2.5 cm depth
SLTPPT_2	Soil texture fraction silt in percent for 10 cm depth
SLTPPT_3	Soil texture fraction silt in percent for 22.5 cm depth
SLTPPT_4	Soil texture fraction silt in percent for 45 cm depth
SLTPPT_5	Soil texture fraction silt in percent for 80 cm depth
SLTPPT_6	Soil texture fraction silt in percent for 150 cm depth
SNDPPT_1	Soil texture fraction sand in percent for 2.5 cm depth
SNDPPT_2	Soil texture fraction sand in percent for 10 cm depth
SNDPPT_3	Soil texture fraction sand in percent for 22.5 cm depth
SNDPPT_4	Soil texture fraction sand in percent for 45 cm depth
SNDPPT_5	Soil texture fraction sand in percent for 80 cm depth
SNDPPT_6	Soil texture fraction sand in percent for 150 cm depth
