# Supplementary material for: Using worldwide edaphic data to model plant species niches: An assessment at a continental extent
Source: PLoS One. 2017 Oct 19;12(10):e0186025. doi: 10.1371/journal.pone.0186025 (PMC5648144; doi:10.1371/journal.pone.0186025)
Supplement: S4 Table — (PDF) [file pone.0186025.s009.pdf]

S4 Table. List of species modeled, number of records (NR), geographic extension (GE), density of records (DR) and standard deviation of the first principal component of climatic variables only (C.SD), climatic and edaphic variables (CE.SD) and edaphic variables only (E.SD).

Species	NR	GE	DR	C.SD	CE.SD	E.SD
Abies guatemalensis Rehd.	46	14108	0.00326	1.129	2.581	3.214
Acacia aroma Hook. & Arn.	159	37983	0.00419	1.150	2.414	2.503
Acacia caven (Molina) Molina	123	34963	0.00352	1.263	3.171	2.962
Acacia furcatispina Burkart	35	15379	0.00228	1.465	3.615	3.737
Acacia praecox Griseb.	75	14282	0.00525	0.787	1.354	1.475
Acalypha macrostachya Jacq.	459	171035	0.00268	1.758	3.052	3.100
Acrocomia aculeata (Jacq.) Lodd. ex Mart.	166	236944	0.00070	1.359	1.439	1.568
Adesmia volckmannii Phil.	63	7715	0.00817	0.624	1.647	1.969
Allagoptera campestris (Mart.) Kuntze	106	38743	0.00274	0.724	1.381	1.517
Anadenanthera colubrina (Vell.) Brenan	330	150711	0.00219	1.247	2.141	2.176
Annona nutans (R.E.Fr.) R.E.Fr.	64	14688	0.00436	0.522	1.262	1.282
Antonia ovata Pohl	113	83016	0.00136	1.524	2.153	1.779
Araucaria angustifolia (Bertol.) Kuntze	48	5915	0.00811	0.625	1.543	2.160
Aspidosperma quebracho-blanco Schltdl.	83	20371	0.00407	0.998	2.048	2.098
Astrocaryum murumuru Mart.	66	63762	0.00104	1.361	1.724	1.338
Astronium graveolens Jacq.	282	205073	0.00138	1.343	1.970	1.970
Atriplex canescens (Pursh) Nutt.	178	34572	0.00515	1.094	1.551	1.709
Attalea butyracea (Mutis ex L.f.) We	104	53507	0.00194	1.270	2.335	2.291
Baccharis crispa Spreng.	142	78551	0.00181	1.296	2.467	2.459
Baccharis gilliesii A.Gray	34	23677	0.00144	1.202	3.284	3.357
Baccharis trimera DC.	162	88715	0.00183	1.473	2.840	2.764
Baccharis ulicina Hook. & Arn.	63	17331	0.00364	1.144	2.837	2.972
Berberis microphylla Forst.	44	15605	0.00282	1.288	2.477	2.397
Brosimum lactescens (S.Moore) C.C.Berg	248	134197	0.00185	1.661	2.207	2.120
Bulnesia sarmientoi Lorentz ex Griseb.	22	3643	0.00604	0.661	1.472	1.521
Byrsonima coccolobifolia Kunth	112	93649	0.00120	1.164	1.388	1.559
Cabralea canjerana (Vell.) Mart.	157	120295	0.00131	2.111	2.614	2.206
Calatola costaricensis Standl.	201	74042	0.00271	1.710	3.213	3.382
Calophyllum brasiliense Cambess.	541	249427	0.00217	1.713	2.513	2.456
Casearia decandra Jacq.	188	165441	0.00114	1.629	2.192	2.029
Cecropia obtusifolia Bertol.	165	52289	0.00316	2.349	3.532	3.110
Cecropia pachystachya Trécul	167	53447	0.00312	0.879	1.270	1.558
Cedrela fissilis Vell.	137	159984	0.00086	1.630	2.365	2.104
Cedrela odorata L.	436	242688	0.00180	1.832	2.704	2.667
Ceiba speciosa (A. St.-Hil.) Ravenna	80	105164	0.00076	1.062	1.662	1.816
Chuquiraga avellanedae Lorentz	35	8281	0.00423	0.594	1.576	1.722
Chuquiraga erinacea D.Don	75	26860	0.00279	0.854	2.280	2.379
Copaifera langsdorffii Desf.	233	81916	0.00284	0.878	1.394	1.544
Cordia trichotoma (Vell.) Steud.	286	68706	0.00416	0.874	1.652	1.795
Cupania vernalis Cambess.	97	69468	0.00140	1.430	2.066	1.738
Curatella americana L.	467	231996	0.00201	1.302	1.924	2.062

Species	NR	GE	DR	C.SD	CE.SD	E.SD
Digitaria californica (Benth.) Henrard	324	214364	0.00151	1.210	2.923	3.225
Digitaria californica (L.) Greene	357	352845	0.00101	1.664	3.390	3.738
Drimys winteri J.R.Forst. & G.Forst.	53	18215	0.00291	1.274	2.917	3.063
Duguetia furfuracea (A.St.-Hil.) Saff.	390	44976	0.00867	0.517	1.428	1.637
Eperua falcata Aubl.	72	29121	0.00247	0.750	1.698	1.653
Eperua leucantha Benth.	29	3770	0.00769	0.705	1.925	2.460
Eragrostis lugens Nees	336	264847	0.00127	1.387	2.862	2.842
Eragrostis mexicana (Hornem.) Link	387	287283	0.00135	2.028	2.607	2.794
Eschweilera coriacea (DC.) S.A.Mori	324	112878	0.00287	1.380	2.158	2.023
Eugenia biflora (L.) DC.	200	158191	0.00126	1.263	2.528	2.474
Euterpe oleracea Mart.	72	93195	0.00077	2.180	2.685	2.089
Euterpe precatoria Mart.	393	105295	0.00373	1.633	2.439	2.291
Ficus insipida Willdenow	480	191450	0.00251	1.953	2.865	2.508
Flourensia cernua DC.	55	7912	0.00695	0.567	1.106	1.259
Genipa americana L.	551	247187	0.00223	1.757	2.510	2.219
Geoffroea decorticans (Hook. & Arn.) Burkart	58	50862	0.00114	1.307	3.467	3.494
Grindelia chiloensis (Cornel.) Cabrera	43	15882	0.00271	0.759	2.082	2.279
Guarea glabra Vahl	238	148227	0.00161	1.848	3.143	2.930
Guazuma ulmifolia Lam.	1054	268022	0.00393	1.559	2.200	2.149
Handroanthus ochraceus (Cham.) Mattos	162	118411	0.00137	0.981	1.588	1.668
Helicostylis tomentosa (Poepp. & Endl.) J.F.Macbr.	248	137789	0.00180	1.651	2.152	1.944
Hevea brasiliensis (Willd. ex A.Juss.) Müll.Arg.	85	138271	0.00061	1.443	2.188	1.999
Hieronyma alchorneoides Allemão	347	173089	0.00200	1.886	3.079	2.749
Inga vera Willd.	670	255898	0.00262	1.465	2.595	2.616
Ipomoea carnea Jacq.	317	305444	0.00104	1.613	2.405	2.629
Iriartea deltoidea Ruiz & Pav.	268	56482	0.00474	1.833	2.620	2.317
Jacaranda copaia (Aubl.) D.Don	315	111133	0.00283	1.480	2.198	2.005
Jatropha dioica Sessé	120	15558	0.00771	0.969	1.889	2.285
Jodina rhombifolia Hook. & Arn. ex Reissek	22	14045	0.00157	0.765	0.800	0.995
Juglans australis Griseb.	29	1996	0.01453	1.230	2.187	1.865
Junellia hookeriana (Covas & Schnack) N. O'Leary & P. Peralta	77	27702	0.00278	0.697	2.776	3.299
Laetia procera (Poepp.) Eichler	167	125701	0.00133	1.349	2.306	2.261
Larrea tridentata (DC.) Coville	350	185224	0.00189	0.915	2.089	2.602
Leptolobium elegans Vogel	207	38145	0.00543	0.621	1.211	1.600
Libidibia paraguariensis (D. Parodi) G.P. Lewis	105	14427	0.00728	0.849	1.667	1.747
Licania apetala (E.Mey.) Fritsch	314	110648	0.00284	1.378	1.881	2.091
Licania heteromorpha Benth.	413	109586	0.00377	1.049	1.747	1.999
Lycium chilense Bert.	97	36039	0.00269	1.112	3.336	3.652
Magonia pubescens A.St.-Hil.	79	37568	0.00210	0.886	1.124	1.144
Matayba elaeagnoides Radlk.	50	66233	0.00075	1.389	1.520	1.772
Menodora integrifolia Steud.	36	34525	0.00104	1.640	3.882	3.524
Metrodorea flavida K. Krause	91	24427	0.00373	0.801	1.223	1.160

Species	NR	GE	DR	C.SD	CE.SD	E.SD
Mulguraea tridens (Lag.) N.O'Leary & P.Peralta	32	6554	0.00488	0.583	1.356	1.870
Mulinum spinosum Pers.	144	19762	0.00729	0.789	1.921	2.368
Nassauvia axillaris (Lag. ex Spreng.) D.Don	73	12052	0.00606	1.027	2.843	3.100
Nothofagus antarctica (G.Forst.) Oerst.	43	11866	0.00362	1.098	2.579	2.773
Nothofagus dombeyi (Mirb.) Oerst.	30	2284	0.01313	0.928	2.763	3.026
Nothofagus pumilio (Poepp. & Endl.) Krasser	30	7369	0.00407	0.812	1.501	1.742
Oenocarpus bataua Mart.	247	78603	0.00314	1.732	2.387	2.064
Panicum bergii Arechav.	124	55094	0.00225	1.238	2.029	1.864
Pappophorum caespitosum R.E.Fr.	83	25162	0.00330	1.269	3.695	3.793
Parthenium incanum Kunth	152	17290	0.00879	0.794	1.312	1.535
Peltophorum dubium (Spreng.) Taub.	124	34822	0.00356	0.615	1.212	1.462
Persea schiedeana Nees	45	8740	0.00515	1.586	2.695	3.580
Phytolacca dioica L	52	120178	0.00043	1.210	1.682	2.262
Pinus caribaea Morelet	61	67763	0.00090	1.393	2.799	2.516
Pinus hartwegii Lindl.	64	12191	0.00525	1.543	2.740	3.206
Poa ligularis Nees ex Steud.	115	25040	0.00459	0.932	3.700	4.383
Podocarpus parlatorei Pilg.	65	6422	0.01012	0.865	1.793	1.769
Posoqueria latifolia (Rudge) Schult.	517	187022	0.00276	1.731	2.931	2.705
Poulsenia armata (Miq.) Standl.	152	81823	0.00186	1.794	2.760	2.919
Prosopis alba Griseb.	75	18926	0.00396	1.108	2.922	2.870
Prosopis glandulosa Torr.	197	35386	0.00557	0.941	2.250	2.788
Prosopis juliflora (Sw.) DC.	178	277420	0.00064	1.907	2.399	2.503
Prosopis kuntzei Kuntze	36	12759	0.00282	1.089	1.872	1.756
Prosopis nigra Hieron.	54	15425	0.00350	0.956	2.200	2.258
Protium heptaphyllum (Aubl.) Marchand	722	139373	0.00518	1.593	2.039	2.079
Qualea grandiflora Mart.	470	71126	0.00661	0.923	1.532	1.640
Qualea parviflora Mart.	477	57324	0.00832	0.907	1.573	1.787
Rhizophora mangle L.	307	291148	0.00105	1.877	2.587	2.414
Ruprechtia laxiflora Meisn.	71	78996	0.00090	1.266	1.717	1.459
Salix humboldtiana Willd.	290	304953	0.00095	1.899	2.956	3.066
Schinopsis brasiliensis Engl.	212	52919	0.00401	0.554	1.780	2.251
Schinus molle L.	96	126841	0.00076	1.490	3.597	3.495
Sebastiania brasiliensis Spreng.	162	62163	0.00261	0.979	1.577	1.523
Senecio filaginoides DC.	68	26260	0.00259	1.331	3.419	3.595
Setaria leucopila (Scribn. & Merr.) K.Schum.	181	122429	0.00148	1.078	2.510	3.070
Sideroxylon obtusifolium (Roem. & Schult.) T.D.Penn.	153	210585	0.00073	1.275	1.733	1.827
Siparuna decipiens (Tul.) A.DC.	255	67669	0.00377	1.321	1.667	1.272
Spondias purpurea L.	319	239915	0.00133	1.671	2.548	2.469
Trema micrantha (L.) Blume	1227	293026	0.00419	1.823	2.703	2.553
Vochysia tucanorum Mart.	299	43243	0.00691	0.575	1.432	1.660
Xylopia aromatica (Lam.) Mart.	462	140568	0.00329	1.450	1.647	1.716
Ziziphus joazeiro Mart.	47	48086	0.00098	0.708	1.391	1.784
